# Host-pathogen interaction profiling of nontypeable *Haemophilus influenzae* and *Moraxella catarrhalis* coinfection of bronchial epithelial cells

**DOI:** 10.1128/msphere.00242-25

**Published:** 2025-06-10

**Authors:** Adonis D’Mello, Timothy F. Murphy, Martina Wade, Charmaine Kirkham, Yong Kong, Hervé Tettelin, Melinda M. Pettigrew

**Affiliations:** 1 Department of Microbiology and Immunology, Institute for Genome Sciences, University of Maryland School of Medicine, Baltimore, Maryland, USA; 2 Division of Infectious Diseases, Department of Medicine, University at Buffalo, The State University of New York, Buffalo, New York, USA; 3 Department of Epidemiology of Microbial Diseases, Yale School of Public Heath, New Haven, Connecticut, USA; 4 Department of Biostatistics, Yale School of Medicine, New Haven, Connecticut, USA; 5 Bioinformatics Resource at the W.M. Keck Foundation Biotechnology Resource Laboratory, Yale School of Medicine, New Haven, Connecticut, USA; 6 Division of Environmental Health Sciences, University of Minnesota School of Public Health, Minneapolis, Minnesota, USA; University of Michigan, Ann Arbor, Michigan, USA

**Keywords:** *Haemophilus influenzae*, coinfection, chronic obstructive pulmonary disease, *Moraxella catarrhalis*, RNA sequencing

## Abstract

Chronic obstructive pulmonary disease (COPD) is a progressive inflammatory lung disease and the third leading cause of death globally. Nontypeable *Haemophilus influenzae* (NTHi) and *Moraxella catarrhalis* (Mcat) are common pathogens in individuals with COPD. Acquisition of NTHi or Mcat can cause acute exacerbations of COPD. NTHi and/or Mcat also persist for months in the lower airways and lead to chronic inflammation. We hypothesized that infection by NTHi or Mcat, singly or during coinfection, requires regulation of specific bacterial and host cell pathways. We investigated this phenomenon using an *in vitro* cell culture model consisting of lung carcinoma H292 cell lines, infected with NTHi, Mcat, or both species. Samples were fractionated into “apical fluid,” containing free-floating bacteria, and adhered/invaded bacteria on or within H292 cells. We used transcriptomic profiling with RNA-seq and various bioinformatic analyses to disentangle host-pathogen interactions in epithelial cell infection from the perspective of each species. Several biological pathways were differentially regulated across all conditions (31, NTHi; 22, Mcat; and 169, human). NTHi transcriptomic profiles differed during mono-infection and coinfection; examples included downregulation of iron-sulfur metabolism (IscR regulon) and differential regulation of quorum sensing in coinfection compared to mono-infection. Mcat was comparatively less affected by the presence of NTHi during coinfection. H292 epithelial cells responded broadly to all infections with distinct responses to mono-infection and coinfection. Enriched host pathways included influenza/interferon/Wnt and proinflammatory responses. These findings suggest common and distinct processes involved in NTHi and/or Mcat-induced COPD pathogenesis and have implications for therapeutic intervention.

**IMPORTANCE:**

Chronic obstructive pulmonary disease (COPD) is a leading cause of death worldwide. Bacteria such as nontypeable *Haemophilus influenzae* (NTHi) and *Moraxella catarrhalis* (Mcat) can cause exacerbations of COPD, and they can persist in the lungs for months, which increases inflammation. We studied how these bacteria interact with lung cells by infecting a cell culture model with NTHi, Mcat, or both. We used RNA sequencing and bioinformatic analysis to examine how the bacteria and host cells respond. When NTHi and Mcat were present together, they behaved differently than when each was alone. We found that different host biological pathways were activated during infection, including those related to inflammation and immune responses. These results provide insights into how NTHi and Mcat contribute to COPD progression and suggest potential targets for new treatments.

## INTRODUCTION

Chronic obstructive pulmonary disease (COPD) is a progressive inflammatory lung disease and the third leading cause of death globally, with 3.23 million deaths in 2019. An estimated 90% of these deaths occur in low- to middle-income countries. Approximately 392 million people aged 30–79 had COPD in 2019 (1). COPD is accompanied by episodes of intermittent worsening of respiratory symptoms, called exacerbations, resulting in significant morbidity and mortality (2). Most exacerbations are caused by bacterial or viral respiratory tract infections (3, 4).

Nontypeable *Haemophilus influenzae* (NTHi) is the most frequently isolated bacteria from the sputum of patients with COPD (5, 6) and accounts for ~30% of acute exacerbations (4, 7, 8). *Moraxella catarrhalis* (Mcat) is the second most common bacterial cause of exacerbations (~10%) followed by *Streptococcus pneumoniae* (9–11). NTHi and Mcat also colonize the upper respiratory tract as commensals (12–15) and cause other infections, including otitis media and pneumonia (6, 8, 10, 16–18).

In addition to causing acute exacerbations, a second important role of bacteria in COPD pathogenesis is chronic lower airway infection (19). In contrast to the healthy human respiratory microbiome, NTHi and Mcat persist for months to years in the lower airways in COPD, including during clinically stable periods (9, 20). These chronic infections cause host inflammatory responses that increase symptoms and accelerate the progressive lung dysfunction that is a hallmark of COPD (21, 22). Respiratory infections in COPD occur as part of a complex microbiome; furthermore, NTHi and Mcat are often present together in the human airways. However, little is known about how these two exclusively human pathogens interact during infection. Unfortunately, there is no validated animal model for COPD that supports colonization and infection with these two pathogens. Moreover, these COPD pathogens do not grow substantially in sputum, making sequencing and downstream transcriptome analysis challenging. To investigate interactions between NTHi, Mcat, and their host, we performed 5-hour mono- and coinfections of human bronchial epithelial cells (National Cancer Institute, NCI-H292 cell line) (23) partially simulating conditions in the airways. Separate assays were performed on supernatant (luminal), adherent, and invading bacteria, simulating cell populations in airways. As part of a prospective study of COPD, we identified individual strains of NTHi and Mcat that caused coinfection in the lower airways of an adult with COPD and studied these strains in detail. Multi-species RNA-seq of H292 cells coinfected with these NTHi and Mcat strains revealed several novel observations, with various biological pathways differentially regulated in all three species. These included multiple micronutrient scavenging in bacterial regulons and bacteria-specific proinflammatory responses in the host epithelium.

**Fig 1:**
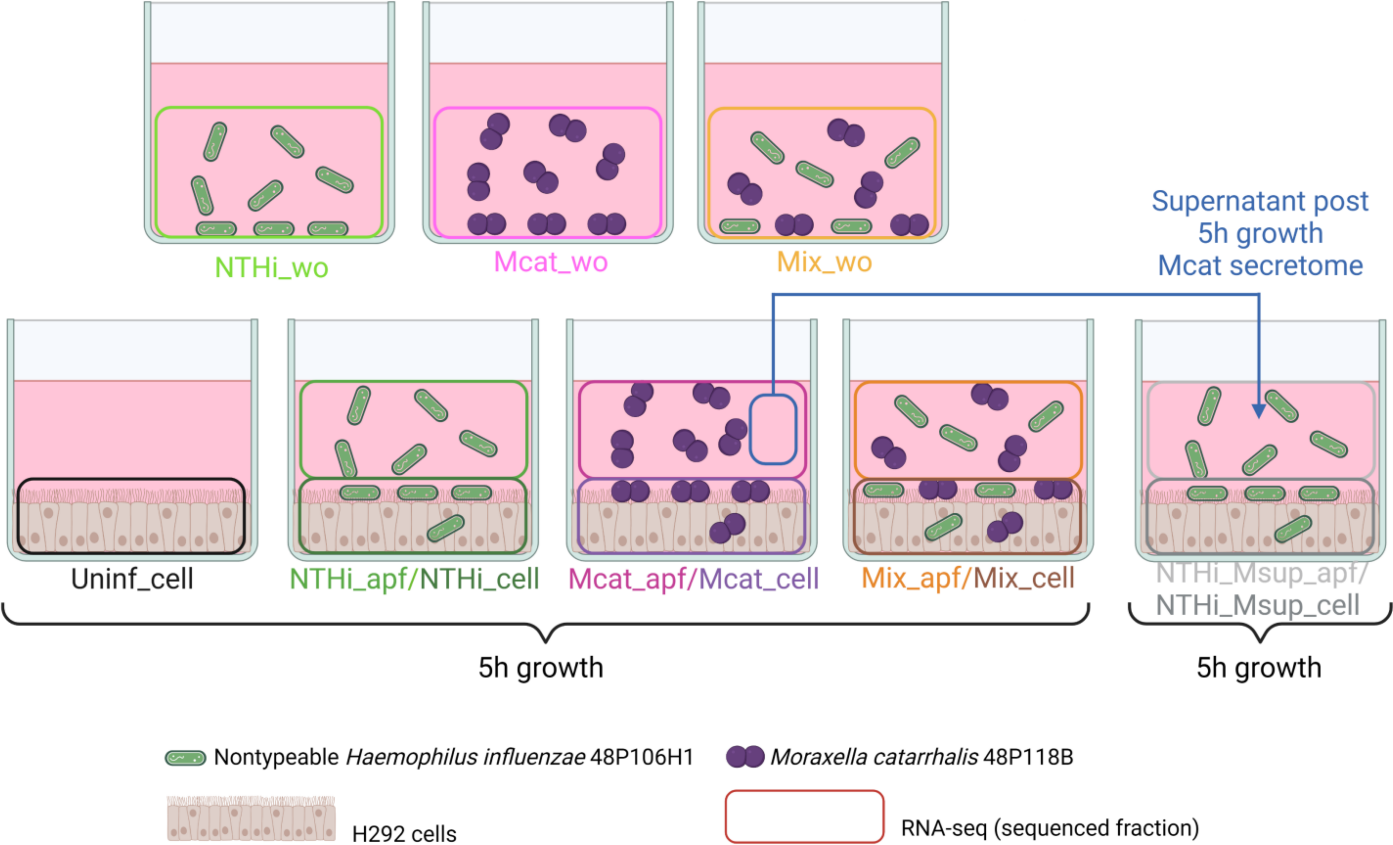
Overview of NTHi and Mcat infection or coinfection of human bronchial epithelial cells (H292) for multi-species RNA-seq. NTHi and Mcat were grown individually or co-cultured in H292 cell culture media in the absence of H292 cells (top three labeled flasks) as bacterial control samples. H292 cells were grown in the absence of bacteria (uninfected control, far left flask), infected individually with NTHi or Mcat, or coinfected (Mix) with both bacteria (bottom four flasks). H292 infected samples had apical fluid (“apf” samples), containing free-floating non-adhered/non-invaded bacteria, and infected H292 cells, with adhered/invaded bacteria on or within H292 cells (“cell” samples), harvested and sequenced separately. Additionally, bacteria-free Mcat apical fluid (Msup) was introduced into NTHi-infected H292 cells to determine Mcat secretory effects (far right flask), referred to as Mcat secretome. All samples were cultured for 5 hours, except NTHi-infected samples with Mcat apical fluid, which had 5 hours of additional growth. Created in BioRender.

Figure 1 shows the study design, in which the NTHi and/or the Mcat isolate(s) was/were incubated in media alone or with H292 cells in an *in vitro* cell culture model (see Materials and Methods). Sample abbreviated names and colors shown in Fig. 1 are maintained in all subsequent figures. Overall, we observed gene regulation patterns of distinct genes and biological pathways for NTHi, Mcat, and human epithelial cells with large transcriptomic differences between NTHi and Mcat during host cell infection compared to growth in laboratory media. NTHi was more sensitive to Mcat’s influence than Mcat was to NTHi’s influence during coinfection. We observed shared host responses to infection by NTHi and Mcat, as well as specific responses to each infecting species. These observations have important implications in understanding coinfection of these two respiratory tract pathogens. These results will be important in guiding the development of novel interventions for the prevention and treatment of bacterial infections in COPD.

## MATERIALS AND METHODS

### Bacterial strains

The NTHi 48P106H1 and Mcat 48P118B strains were isolated from the sputum of an adult with severe COPD enrolled in a 20-year prospective study of adults with COPD, conducted at the Buffalo Veterans Affairs Medical Center (5, 9, 24).

### *In vitro* cell culture models

Three independent sets of flasks were prepared for each condition as shown in Fig. 1. NCI-H292 cells were grown to confluence in culture media. For the *in vitro* cell culture assays, confluent H292 cells were inoculated with 2 mL washed bacteria (2 mL of NTHi or 2 mL Mcat or 1 mL each for the mix). Similarly, for the media-alone assays (wo), culture media was inoculated with 2 mL washed bacteria. The NTHi with Mcat apical fluid (Msup, henceforth referred to as Mcat secretome) flasks were prepared with minor modifications (see Supplemental Methods). Flasks containing infected H292 cells were fractioned into “apical fluid” (apf), containing free-floating non-adhered/non-invaded bacteria, and adhered/invaded bacteria on or within H292 cells (cell). The fractions, as well as media-alone assays, were transferred to RNAprotect (Qiagen, Valencia, CA).

### RNA extraction and sequencing

RNA was extracted using the Maxwell 16 LEV simplyRNA Tissue Kit with modifications, followed by DNase treatment, and purified using the Qiagen RNeasy Mini Elute Clean-up kit. One microgram of purified RNA was sequenced on the Illumina NovaSeq 6000 (100 bp paired-end), after rRNA depletion.

### Transcriptomics and pathway analysis

FASTQ files were mapped to their respective genomes using HISAT (25)/Bowtie2 (26). Gene expression counts were estimated using HTseq (27) followed by batch effect correction using CombatSeq (28) and estimation of normalized expression values and differentially expressed (DE) genes using DESeq2 (29). Weighted correlation network analysis (WGCNA) was performed using the R package WGCNA (30), with DE genes for each species having an average variance stabilizing transformation (VST) count ≥7, using a soft power of 9, and default parameters. Pathway enrichments were performed using ShinyGO (31) on the Kyoto Encyclopedia of Genes and Genomes (KEGG [32]) database for humans, and on KEGG, RegPrecise (33), and Virulence Factor Database (34) for bacteria.

## RESULTS

### Bacterial and human transcriptional profiles reflect major differences between mono-infection and coinfection

Bacterial and human transcriptional profiles were assessed from H292 cells infected with NTHi and Mcat individually and in coinfection (Mix; Fig. 1). To ensure sufficient bacterial RNA-seq data, we performed transcriptomic quality control using rarefaction curves (Fig. S1) for reads mapped to the genomes of both species (accessions: NTHi. CP020006; Mcat, CP158365). The rarefaction curves indicated sufficient sequencing depth for downstream analysis of bacterial gene expression profiles (see Supplemental Material). A comprehensive summary of read mapping statistics and raw read counts is provided in Table S1.

**Fig 2:**
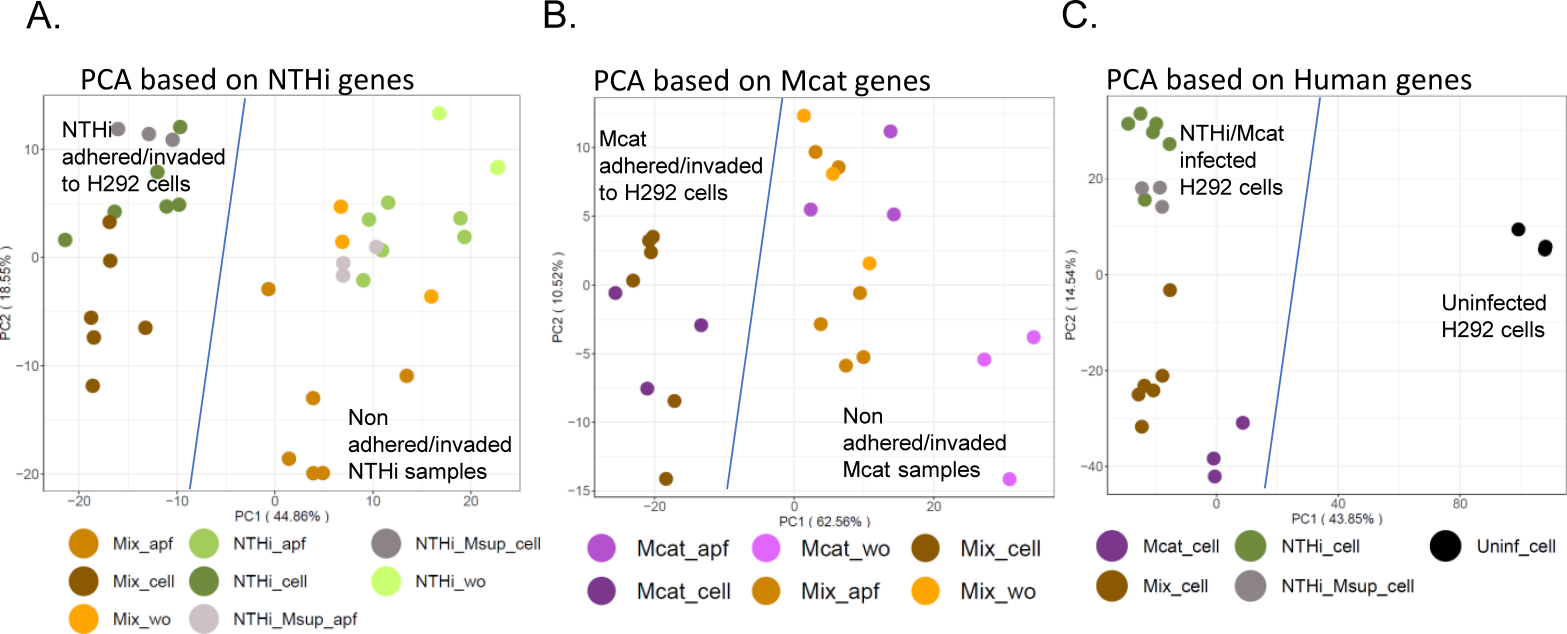
PCA of NTHi, Mcat, and H292 samples. (**A**) PCA of NTHi samples. PC1 (~45% of the variance) separates the infection conditions with NTHi adhered/invaded to H292 cells (cell) on the left and apf or individual NTHi samples (NTHi) on the right. PC2 (19%) partially separates coinfection (Mix) samples (bottom, contain Mcat) from just NTHi samples (top). (**B**) PCA of Mcat samples. PC1 (63%), similar to NTHi, separates Mcat adhered/invaded to H292 cells (left) from apf or individual Mcat samples (right). PC2 (11%) shows no clear trend. (**C**) PCA of H292 samples. PC1 (~44%) separates all infected cells (left) from uninfected cells (right). PC2 (~15%) separates each different infection from top to bottom. Normalized expression values (VST counts) used in each PCA are provided in Tables S2 to S4.

Based on these bacterial read proportions, after 5 hours of mono-infection, Mcat exhibited approximately twice the survival rate of NTHi (percent mapped reads in Mcat_cell vs NTHi_cell), though both species adhered and invaded to similar extents (estimated from approximately equal amounts of read proportions in the apical fluids Mcat_apf vs NTHi_apf). During coinfection, NTHi showed about 25% increased adherence, invasion, and survival compared to Mcat (mix_cell). However, Mcat showed about 50% decreased adherence, invasion, and survival compared to NTHi from coinfection apical fluid (mix_apf). Additionally, Mcat replicated approximately twice as much as NTHi in media alone (mix_wo). Interestingly, Mcat apical fluid enhanced NTHi survival in mono-infection by around 33% (NTHi_Msup_cell vs NTHi_cell), while adherence and invasion rates remained similar between the species (NTHi_Msup_apf vs NTHi_apf). Although actual bacterial proportions were not measured, these results suggest that coinfection affects host/bacterial behavior and survival, and both bacterial species exhibit complex interactions under these conditions. Principal component analysis (PCA) of transcriptional profiles for each species revealed clustering of sample replicates and identified changes in responses across conditions. For NTHi samples (Fig. 2A), PC1 (~45% variance) indicated that the NTHi response is heavily influenced by H292 cells; NTHi and NTHi + Mcat (Mix) infected H292 cells are on the left, and NTHi grown in cell culture medium (wo) or non-invaded/non-adhered bacteria in apf are on the right. PC2 (~19%) reveals some separation of coinfected samples from NTHi alone. Mix sample types (cell, apf, and wo) cluster away from their respective NTHi counterparts, suggesting a significant impact of Mcat on NTHi in all sample types. NTHi samples exposed to Mcat secretome (i.e., Msup) closely cluster with respective unexposed NTHi samples, suggesting only a minor impact of the Mcat secretome on the NTHi response.

Similarly, for Mcat samples (Fig. 2B), PC1 (~63%) revealed that the Mcat response to adherence/invasion of H292 cells is different from Mcat alone or in the apical compartment. However, unlike the NTHi PCA (Fig. 2A), no clear separation was seen between Mcat and Mcat + NTHi (Mix) infected H292 cells, suggesting that Mcat is less affected by the presence of NTHi during H292 coinfection. PC2 (~11%) partially separates Mix_apf samples, and both PCs cluster Mcat_wo samples separately. Given that Mcat_apf samples cluster more closely with Mix_wo/apf samples, this suggests that Mcat responds to both species in a similar manner when in a planktonic state or has limited interactions with host cells.

For H292 cellular responses (Fig. 2C), PC1 (~44%) showed that all bacteria-infected samples were distinct from uninfected samples, as expected. Strikingly, there was separation observed among infection conditions, with Mcat infections closer to uninfected samples than NTHi-containing samples, implying that Mcat infection induces a somewhat blunted response from H292 cells compared to NTHi. PC2 (~15%) separates Mcat-infected samples (bottom half) from NTHi-infected samples (top half). While all NTHi-containing samples are in the same vertical plane (along PC2), NTHi infections exposed to Mcat secretome form their own cluster, implying a stronger response of H292 cells to the presence of NTHi and the Mcat secretome relative to NTHi alone.

### NTHi, Mcat, and human H292 cells differentially regulate specific gene sets during adhered and invaded conditions

**Fig 3:**
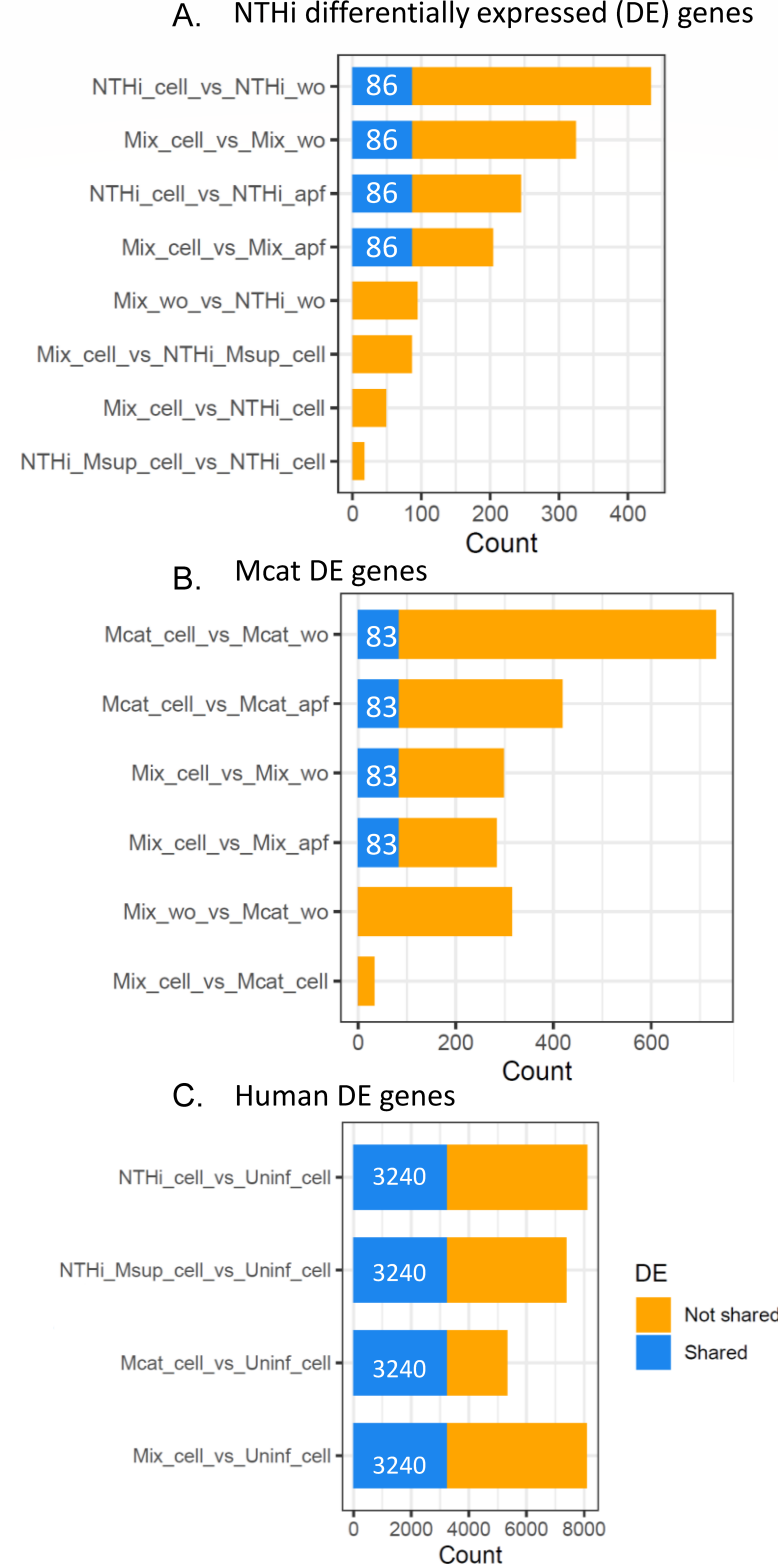
Differential expression analysis of NTHi, Mcat, and human genes. (**A**) Counts (number) of NTHi DE genes across various comparisons. Eighty-six genes were DE across samples with H292 cells vs those without. (**B**) Counts of Mcat DE genes across various comparisons. Eighty-three genes were found to be DE across samples with H292 cells vs those without. (**C**) Counts of human DE genes across infected vs uninfected samples. A total of 3,240 genes were shared across all four comparisons. Lists of all DE genes for each comparison for each species are provided in Table S2 (NTHi), Table S3 (Mcat), and Table S4 (human).

We identified DE genes for all species across growth conditions/fractions. Several NTHi genes were DE (Fig. 3A) in all H292 adhered/invaded cells relative to media alone, implying a robust response to direct contact with H292 cells. However, relative to apical fluid bacteria, fewer DE genes were observed. Of these four comparisons, 86 NTHi genes were shared across all H292 adhered/invaded cells and not among the other sample types (apf and wo, Fig. 3A, blue bar of shared genes).

Adhered/invaded Mcat on H292 cells alone had ~70% more DE genes than NTHi alone (Fig. 3B), indicating a robust Mcat response to contact with H292. For Mcat, 83 genes were shared across all H292 adhered/invaded cells and not among the other conditions.

Lastly, for H292 DE genes, all infected conditions were compared to uninfected H292 cells. Thousands of H292 genes were DE across all infection conditions, with similar amounts for NTHi infection and NTHi/Mcat coinfection (Fig. 3C). Interestingly, Mcat infection regulated ~30% fewer H292 DE genes than the other conditions, suggesting a blunted response of H292 cells, a pattern that was also observed on the H292 PCA (Fig. 2C). Overall, 3,240 H292 genes were DE across all infection conditions. DE gene lists and overlaps across conditions are provided in Table S2 (NTHi), Table S3 (Mcat), Table S4 (H292), and Fig. S2.

We performed WGCNA-based clustering of the normalized expression values of all DE genes (after filtering; see Materials and Methods) for each species to identify specific gene expression patterns that correlated with certain sample combinations (e.g., NTHi_cell, Mcat_cell, etc.). Multiple gene expression modules, defined as subsets of DE genes with similar expression profiles, were detected for each species.

**Fig 4:**
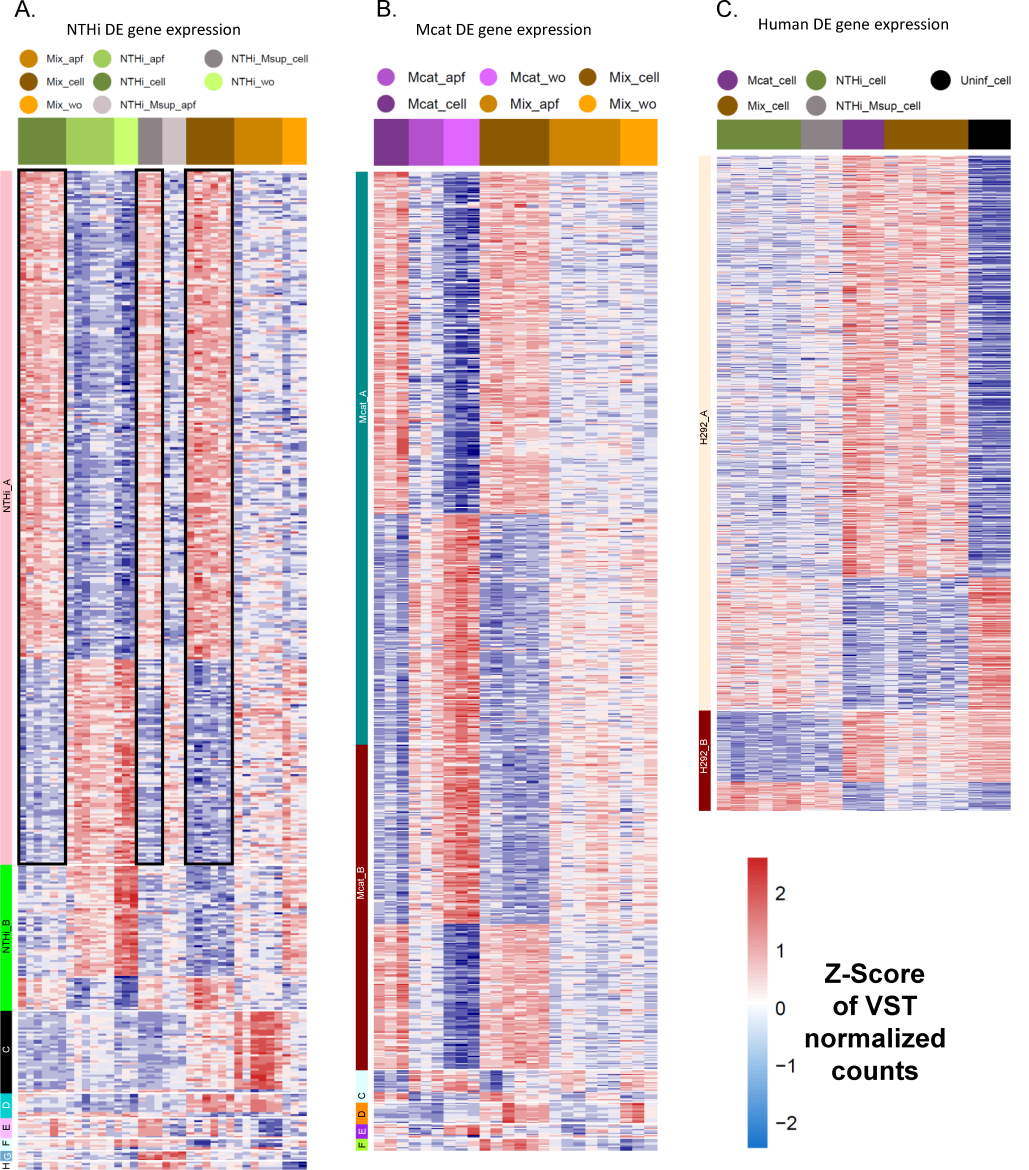
Condition-specific gene expression patterns of NTHi, Mcat, and human DE genes. (**A**) WGCNA heatmap of normalized expression levels (VST counts) of NTHi DE genes showing eight modules (NTHi_A–NTHi_H) which correlated with specific conditions. Black boxes show NTHi gene expression patterns associated with epithelial cell infection. (**B**) WGCNA heatmap of normalized expression levels of Mcat DE genes showing six modules (Mcat_A–Mcat_F) which correlated with specific conditions. (**C**) WGCNA heatmap of normalized expression levels of human DE genes showing two modules which correlated with Mcat infected (H292_A) and NTHi infected (H292_B) samples. A list of DE genes in each module is provided in Table S5 (NTHi and Mcat) and Table S6 (human).

For NTHi (Fig. 4A), the largest module consisted of 337 genes and was directly associated with NTHi invaded/adhered to H292 cells, regardless of the presence of Mcat secretome or Mcat coinfection (module NTHi_A). Genes in this module that conformed to the same expression pattern in all samples with H292 cells (NTHi_cell, NTHi_Msup_cell, and Mix_cell) are enclosed in black boxes to exemplify similar expression trends. There were seven other modules (NTHi B through H) that also correlated with certain sample types for NTHi. Module NTHi_B harbored genes with enhanced regulation for NTHi individual culture in media (NTHi_wo). Module NTHi_C primarily contained upregulated genes specific to NTHi apical fluid during co-culture on H292 cells (Mix_apf). Module NTHi_D consisted of genes specific to coinfection of H292 cells at any stage of H292 infection (Mix_cell and Mix_apf). Module NTHi_E was selected for NTHi genes responsive to Mcat secretome only during invasion/adhesion (NTHi_Msup_cell) and not during apical survival (NTHi_Msup_apf). Module NTHi_F highlighted genes that are downregulated during coinfection of H292 cells at any stage of H292 infection (Mix_cell and Mix_apf), suggesting that Mcat presence supplements their usage. Modules NTHi_G and NTHi_H consisted of NTHi genes that were particularly sensitive to the Mcat secretome (NTHi_Msup_cell/NTHi_Msup_apf).

Fewer modules of regulated genes were detected for Mcat compared to NTHi (Fig. 4B). Two modules, Mcat_A and Mcat_B, reflected gene expression changes driven by Mcat in individual culture in media (Mcat_wo, similar to module NTHi_B). Similar to module NTHi_A, Mcat modules Mcat_A (444 genes) and Mcat_B (252) were directly associated with Mcat invaded/adhered to H292 cells and not apf (Mcat_cell and Mix_cell), regardless of the presence of NTHi in coinfection. Module Mcat_C genes are also partially associated with Mcat invaded/adhered to H292 cells and not apf, albeit with variation among some replicates (Mcat_cell and Mix_cell). Module Mcat_D and Mcat_F showcased small gene sets that were upregulated by Mcat during invasion/adherence during coinfection (Mix_cell). Lists of all the genes in NTHi and Mcat modules are in Table S5.

Five major modules were detected for H292 cells. Three of the five modules (not shown) consisted of genes that were differentially regulated across all infection conditions relative to uninfected H292 cells; these modules did not clearly distinguish gene expression patterns specific to NTHi, Mcat, or mixed infection. However, two modules H292_A and H292_B did highlight gene expression patterns specific to Mcat presence (Mcat_cell and Mix_cell), as well as specific to NTHi presence alone (NTHi_cell and NTHi_Msup_cell), respectively (Table S6).

### NHTi and Mcat coinfection identify specific micronutrient scavenging pathways and environmental adaptations for both species

**Fig 5:**
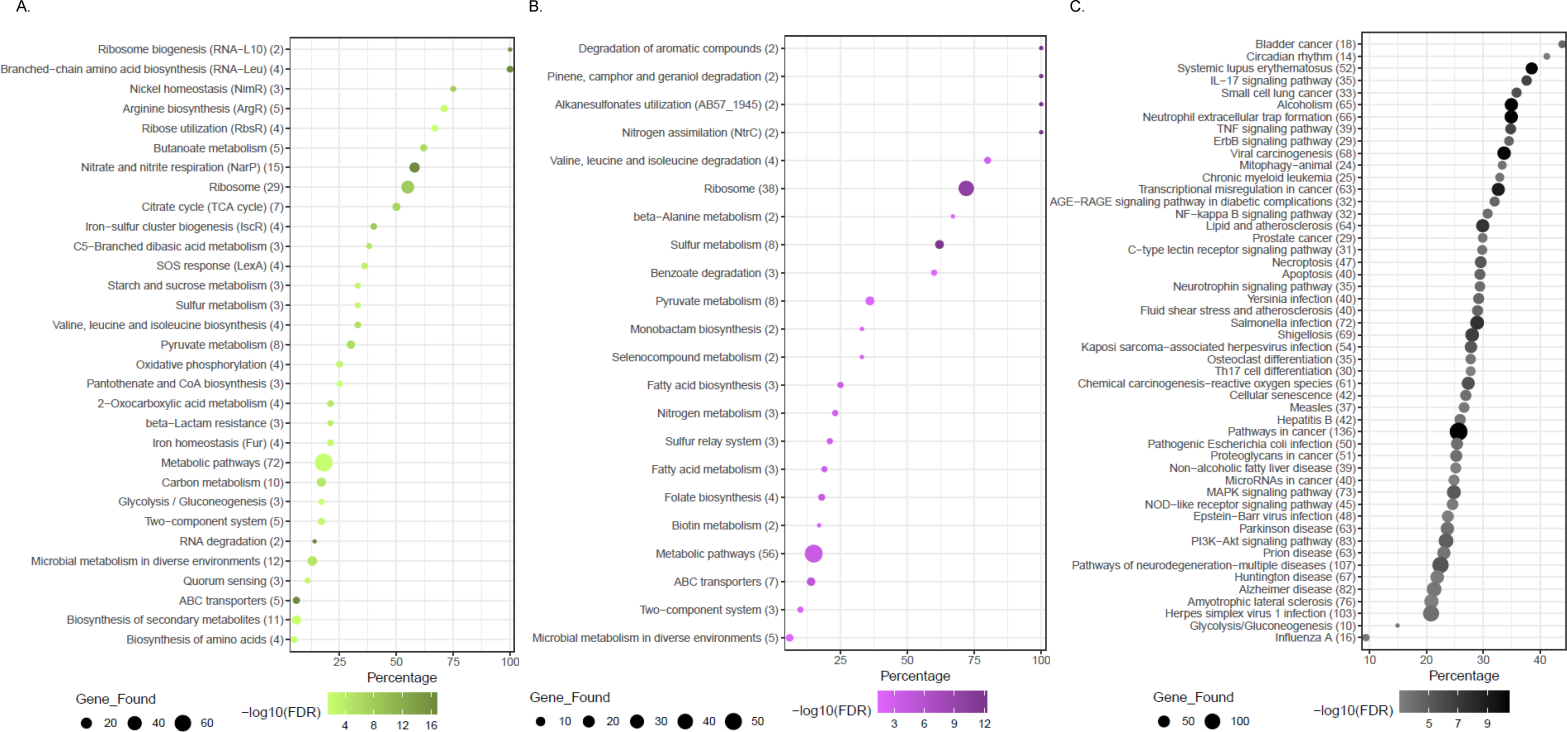
Pathway enrichment analysis based on NTHi, Mcat, and human DE genes. *X* axes represent the percentage of detected genes in the pathway, and *Y* axes represent the enriched pathways and the number of genes that were detected. Percentage indicates the fraction of DE genes detected relative to all genes of a pathway. (**A**) Enrichment statistics of all significantly enriched pathways of NTHi DE genes (KEGG and RegPrecise databases). (**B**) Enrichment statistics of all significantly enriched pathways of Mcat (KEGG and RegPrecise). (**C**) Enrichment statistics of all significantly enriched pathways of human DE genes (KEGG via ShinyGO). Expanded lists of enriched pathways are provided in Table S5 (NTHi and Mcat) and Table S6 (human).

Next, we performed pathway enrichment analyses for all the modules for each species against three pathway databases. For NTHi, multiple pathways (Fig. 5A) were significantly enriched across the eight modules in Fig. 4A. Notable pathways with strong gene expression profiles are exhibited as heatmaps in Fig. S3 alongside the equivalent Mcat pathway with its respective genes. One such example was iron-sulfur metabolism in NTHi through the regulon of transcriptional regulator IscR (locus tag BV083_495). Nearly all genes were downregulated in coinfection with Mcat on H292 cells (Fig. S3A), implying a reduced requirement for iron-sulfur metabolites during coinfection. NTHi is also capable of sulfur metabolism through other genes such as dimethyl sulfoxide reductases A, B, and C (Fig. S3B; BV083_1176, BV083_1177, and BV083_1179). These likely had a role only during coinfection at the H292 cell surface in the apical fluid but not as much during adherence or invasion during NTHi and Mcat coinfection. Moreover, these *dmsABC* genes are part of a larger NarP regulon (BV083_858, a LuxR-family regulator), consisting of multiple operons related to nitrite/nitrate-based respiration (Fig. S3C), all of which follow the same pattern of gene expression, again implying a role only during coinfection at the H292 cell surface in the apical fluid. The primary differentiating factor between NTHi infection and coinfection was the NTHi oligopeptide transporter operon *oppABCDF* (BV083_1251–BV083_1255), which is involved in quorum sensing (Fig. S3D) and beta-lactam resistance (Fig. S3E). The *oppABCDF* operon upregulation suggests uptake of signaling peptides secreted by Mcat but only during coinfection and not in media. As this signal was not seen in NTHi_Msup_cell samples, this observation suggests that uptake of these peptides is required during coinfection but not during individual Mcat infection.

Fewer pathways were identified in the Mcat modules of DE genes compared to NTHi (Fig. 5B). The most prominent pathway for Mcat infection and coinfection involved sulfur metabolism. Sulfur metabolism in Mcat is largely controlled by *cys* genes involved in cysteine biosynthesis (35) and H_2_S production (36), all of which were highly upregulated during coinfection (Fig. S3B) and partially during coculture with NTHi. This observation suggests that coinfection with NTHi results in sulfur-depleted conditions for Mcat or the induction of reactive sulfur species in the environment (36). Another pathway that showcased genes preferred during coinfection was the folate biosynthesis pathway (Fig. S3F). A subset of genes within this pathway is upregulated primarily in coinfection, namely *moaE*, *moaC*, *mobA*, *moaB*, and *moaA* (AABM16_03845, AABM16_03490, AABM16_03500, AABM16_03505, and AABM16_03515, respectively). Genes *moaE*, *moaC*, and *mobA* are part of a four-gene operon involved in molybdenum homeostasis in multiple species (RegPrecise database [33]). The missing *moa* gene was annotated as a pseudogene in this Mcat strain and hence is not shown. However, all five genes listed are in the same locus. A requirement for molybdenum can occur alongside increased redox activity in sulfur metabolism (37), which is likely the case here. One other pathway that showed gene expression specific to Mcat infection and coinfection conditions was lipooligosaccharide (LOS) biosynthesis (Fig. S3G), although it was not significantly enriched among the Mcat modules. Several *lpx* genes that are upregulated when Mcat adheres or invades H292 cells were identified, implying a need for the LOS to allow adherence and invasion of H292 cells.

Subsets of the bacterial pathways are described above. The complete pathway results for NTHi and Mcat are provided in Table S5. Heatmaps of all bacterial pathways are also provided in a Figshare repository (https://doi.org/10.6084/m9.figshare.27963945).

### Human pathways responsive to mono- and coinfections

We performed pathway enrichments for H292 DE genes from the WGCNA modules (Fig. 4C) using ShinyGO (31) against the KEGG database (32). Module H292_A, encompassing gene expression patterns specific to the presence of Mcat (Mcat_cell and Mix_cell), enriched 55 pathways, while module H292_B genes, specific to NTHi presence, enriched only six pathways (Table S6), as it had much fewer genes. The remaining modules together enriched 143 pathways. The top 50 most enriched pathways across all H292 modules are shown in Fig. 5C. Some of these reflected general gram-negative bacterial infection pathways, such as *Yersinia*, *Salmonella*, *Shigella*, and *Escherichia coli* infection, which are also likely affected by Mcat and/or NTHi. We also observed activation of various cytokine (IL-17, TNF, etc.) signaling and immune cell (Th1, Th17, etc.) differentiation pathways. There were also a few viral infection pathways, such as influenza, hepatitis B, etc. Among the pool of pathways that were enriched, several had no obvious relation to our infection model, such as a handful of cancer pathways (e.g., leukemia) and human diseases such as Parkinson’s. As such, we chose to further investigate specific pathways clearly related to infection conditions pertinent to our experimental design, or those enriched by Mcat- and NTHi-specific modules, H292_A and H292_B, respectively, while recognizing that some unexpected pathways may be relevant after further investigation.

To better understand the infection-specific gene expression profiles of some of the enriched pathways, we overlaid expression heatmap data directly onto the respective KEGG pathways (Fig. S4 includes non-DE genes, see Materials and Methods). The enriched pathway of bacterial invasion of epithelial cells (Fig. S4A) reveals upregulation of integrin ITAG5 and SEPTIN only in Mcat infection or Mcat secretome conditions. This suggests that Mcat can interact with ITAG5 at the epithelial cell surface and may lead to downstream actin polymerization via Septin (38), allowing endocytosis and invasion by Mcat.

A highly enriched pathway within the H292_A module (Mcat associated) was influenza A infection (Fig. S4B), revealing upregulation of transmembrane serine protease 11D (TMPRSS11D) primarily during Mcat infection or coinfection but not when cells were exposed to the Mcat secretome. This suggests the direct involvement of Mcat leading to the activation of TMPRSS11D. Many other genes in this pathway had similar patterns of expression owing to direct Mcat involvement, such as two interferon-related genes (IFNA2 and IRF7) and other chemokines such as CCL5, CXCL10, and IL12A. Other genes in this pathway were also upregulated among all infected samples, including NF-κB, CXCL8, and CCL2, suggesting that NTHi infection alone can trigger the influenza A infection pathway. However, Mcat’s presence further exacerbates the pathway and its downstream effects via different genes.

While the H292_B module (NTHi-associated) had only six pathways enriched, two genes related to Wnt signaling were involved in nearly all six pathways, WNT2B and FZD10. Overlaying gene expression on the Wnt signaling pathway (Fig. S4C) showed downregulation of WNT2B/WNT1 (synonymous in function in this pathway) and other WNT genes such as WNT11 and WNT5A. Other genes exhibiting potentially NTHi-specific gene regulation included JUN, FOSL1, RUVBL1, and ZNRF3 (upregulation with NTHi alone) and DKK, APCDD1, and PRICKLE (downregulation in NTHi-infected/coinfected samples). Here, as well, many non-module genes had expressions associated with Mcat infection/coinfection alone (such as RAC, FZD5, etc.).

Looking at broader bacterial infection pathways such as Toll-like receptor signaling (Fig. S4D), we observed TLR2 and TLR4 upregulation in Mcat-infected samples and NTHi-infected samples with Mcat apical fluid, likely triggered by Mcat surface LOS or lipoproteins. Upregulation of other factors was also correlated with Mcat-infected samples such as TICAM2 and IRF7, leading to the activation of potentially Mcat-specific cytokines including IFNA2, CCL5, CCL3, IL12A, CXCL10, and CXCL11. Similarly, NTHi also influenced the upregulation of TLR6, JUN, and specific cytokines TNF, IL1B, and IL6. Ultimately, many of the same cytokines were affected by both NTHi and Mcat, but often to different degrees.

Upon examination of epithelial tight junction pathways (Fig. S4E), specific responses were triggered by each infecting species. Mcat-induced upregulation of claudin (CLDN4), which could result in decreased paracellular permeability, suggests that epithelial cells resist paracellular invasion during Mcat infection, but not NTHi infection. Similarly, the presence of NTHi-induced upregulation of occludin (OCLN) and MYH9 possibly improves cellular polarity as a resistance mechanism to NTHi infection.

## DISCUSSION

**Fig 6:**
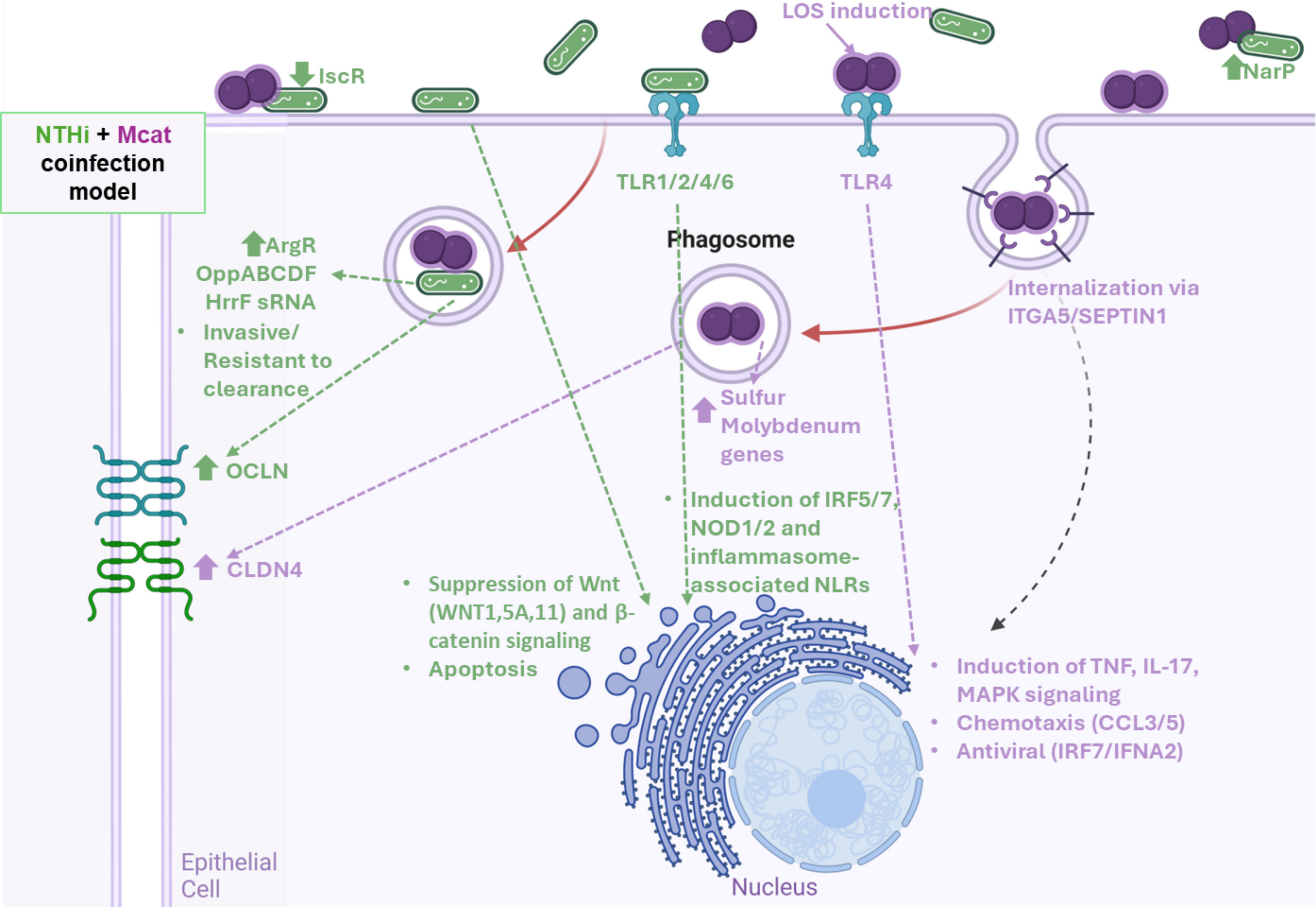
Proposed model of NTHi and Mcat coinfection. Green arrows and text represent NTHi-induced changes or host responses to NTHi. Purple arrows and text represent Mcat-induced changes or host responses to Mcat. Up or down arrow glyphs indicate gene up or downregulation identified in this study. Bullet points indicate enriched gene sets or pathways. Abbreviation: NLRs, nucleotide-binding oligomerization domain-like receptors. Created in BioRender.

We present gene regulation patterns of biological pathways in NTHi, Mcat, and the human host during mono-infection and coinfection of bronchial epithelial cells, revealing differential regulation across species and conditions. NTHi, Mcat, and H292 cells differentially regulate specific gene sets when bacteria are planktonic in apical fluid relative to during adherence and invasion. NTHi transcriptomic profiles that differed during mono-infection and coinfection included genes related to iron, quorum sensing, and arginine. Genes related to sulfur metabolism and molybdenum were upregulated in Mcat during coinfection. However, Mcat was comparatively less affected by the presence of NTHi during coinfection (evidenced by modules NTHi_B, C, and D: 122 DE genes; Mcat_D and F: 27 DE genes). Human host modules H292_A and H292_B, which are related to influenza infection, Wnt signaling, and Toll-like receptor signaling, were also differentially responsive to the type of bacterial infection. Figure 6 shows a proposed model for NTHi and Mcat coinfection of human bronchial epithelial cells that is based on our data in combination with published data from others.

The NTHi IscR regulon (Fig. S3A) was downregulated specifically during coinfection of host cells (adhered/invaded and apical surface compartments). IscR is involved in Fe-S homeostasis, oxidative and nitrosative stress (39), and virulence in other pathogens (40). It is possible that during coinfection, changes in Fe-S availability and responses to oxidative/nitrosative stress by Mcat caused the downregulation of NTHi IscR genes. Another example unique to the apical surface is the upregulation of the NarP regulon (Fig. S3C) in the presence of Mcat. The production of host reactive nitrogen species secreted (41) into the microenvironment upon detection of lipooligosaccharide (42), with increased host signaling molecules such as TNFα (42), might explain this NTHi defensive response of nitrogen-based respiration. Metabolic changes only occurring in coinfection, along with Mcat being relatively unperturbed by NTHi, indicate that Mcat impacts NTHi and the microenvironment via non-secreted factors.

Upregulation of the *oppABCDF* operon in NTHi during coinfection affected quorum sensing and beta-lactam resistance (Fig. S3D and E). This has been observed previously in NTHi-Mcat biofilms associated with otitis media. Mcat was responsive to NTHi quorum-sensing molecule AI-2, and NTHi-Mcat biofilms were more resistant to antibiotics (43). The *oppABCDF* operon is present in several bacteria (44, 45) and promotes intracellular survival. We have previously shown reduced persistence of an *oppA* knockout mutant in Mcat in a pulmonary clearance model (45). These findings suggest that NTHi *oppABCDF* upregulation is a transition away from biofilm to invasiveness during coinfection, along with increased resistance to host clearance.

Other studies demonstrated the importance of arginine for NTHi growth and survival with/without competing microbes (46, 47). We observed a similar phenomenon during NTHi mono-/coinfection with strain 48P106H1 (Fig. S5A). Besides this, the fur (ferric uptake regulator) regulon involved in iron homeostasis has an important role in otitis media pathogenesis (48, 49). Fur is capable of gene activation or repression, sometimes indirectly via a small RNA (sRNA) called HrrF (48). We observed the expression of HrrF primarily in NTHi mono-/coinfection samples on epithelial cells, as well as condition-specific regulation of the fur regulon (Fig. S5B and C). Only a single copy of HrrF was present in NTHi, and no ortholog was found in Mcat.

One study of NTHi sputum samples from patients with pneumonia (similar to our NTHi_sup vs NTHi_wo comparison) showed downregulation of the tricarboxylic acid (TCA) cycle (NTHi_KEGG_Pathway_00020; see https://doi.org/10.6084/m9.figshare.27963945) and cytochrome c biogenesis genes (*ccm* genes, NarP regulon; Fig. S3C) (50), as well as upregulation of purine regulon genes (PurR regulon; see https://doi.org/10.6084/m9.figshare.27963945) (50, 51) when comparing NTHi during pneumonia vs in culture. We observed that all these processes are relevant to coinfection, with operons from these pathways largely upregulated at the apical surface of the epithelium.

Upregulation of sulfur metabolism genes in Mcat during coinfection (Fig. S3B) could be a defensive response to the epithelium during coinfection. Bacteria produce H_2_S and other sulfur-based ions with these genes (36), enabling growth, antibiotic resistance (52, 53), resistance to immune-mediated killing (54), and even anaerobic respiration in biofilms (55). Molybdenum uptake genes were also identified as relevant to coinfection (Fig. S3F). Both these systems were involved in Mcat interactions with Detroit-562 cells in a microarray study (37). Together, these data suggest that the need for these systems increases with NTHi presence. Interestingly, we also observed upregulation of LOS biosynthesis genes by Mcat during mono-/coinfection of H292 cells (Fig. S3G). This was not observed for NTHi. Further exploration of genes related to lipid A or LOS (Fig. S6) revealed the same consistent upregulation of these genes for Mcat but not NTHi, demonstrating the involvement of Mcat LOS during mono-/coinfection of epithelial cells. Other NTHi/Mcat genes and pathways of interest are available in Tables S2, S3, and S5 and at https://doi.org/10.6084/m9.figshare.27963945.

The roles of bacterial iron-sulfur, nitrite/nitrate, or molybdenum metabolism in COPD pathogenesis are potentially linked in both species. Consider the upregulated Mcat molybdenum genes *moaA/C/E*, *moaB,* and *mobA*. Together, these enzymes form a series of reactions culminating in the production of a guanylyl molybdenum cofactor (KEGG: folate biosynthesis*—M. catarrhalis* mcat00790). This cofactor is thought to be essential for nitrate reductases in *E. coli* (56) and dimethyl sulfoxide reductases (upregulated in NTHi, *dmsABCDX*-NarP regulon, and KEGG: sulfur metabolism*—H. influenzae* hin00920) in NTHi (57). These *dms* reductases are also linked to iron-sulfur cluster IscR (58). IscR is a crucial regulator in bacterial pathogenesis, particularly in species like *Vibrio vulnificus*, *Yersinia*, and *Pseudomonas aeruginosa* (59, 60). IscR is known to trigger the expression of virulence factors that enable invasion and infection. For example, in *Yersinia pseudotuberculosis*, IscR directly regulates the type III secretion system that contributes to virulence (61). H292 cells are also known to produce reactive species and increased mucin on infection by NTHi, Mcat (62), and *Pseudomonas* (63). It is possible that this cofactor is shared by Mcat to NTHi during coinfection, allowing each species to use its respective sets of enzymes to better survive the impact of reactive species released during coinfection. Observing consistent upregulation of NTHi IscR in mono-infection, but not coinfection with Mcat, implies a reduced need for these genes as Mcat handles a part of the infection burden. However, metal ions Fe^2+^ and Mo^2+^ appear important to both species in their handling of host reactive species.

We have previously investigated gene conservation in several clinical isolates of NTHi (270 isolates, from 78 patients) and Mcat (69, from 21 patients), including our assayed strains, from a reverse vaccinology perspective (64). These isolates encompassed 77 sequence types over a period of 15 years (24). We observed that the genes within the pathways we prioritized for both species are highly conserved within their respective populations. For example, in Mcat, genes in the sulfur metabolism and LOS biosynthesis pathway were present in 98–100% of these Mcat clinical isolates. For folate biosynthesis, the genes *moeA*, *moaE/C*, *mobA*, and *moaB/A* were present in ~90% of isolates, with the remaining genes present in ~100% of isolates. Similarly, in the case of NTHi, most genes that we prioritized from our enriched biological pathways were very highly conserved. For regulons ArgR and IscR, all genes were conserved (98–100%). Minor variability in genes of the NarP (92–100%) and Fur regulons (98–100%, *nrf* operon: 94%) was found. Gene conservation for quorum sensing and beta-lactam resistance was ~96–100%, with the *oppABCDF* operon present in ~98% of the isolates. Given the high conservation (~90% or higher) of these pathways and their strict regulation in gene expression, these findings are likely representative of clinical infection conditions and will generally apply to most other strains in COPD infections.

Prior studies have examined the host response to COPD pathogens. An examination of transcriptomic profiles of A549 human lung adenocarcinoma epithelial cells when infected with macrolide-resistant or susceptible Mcat strains revealed upregulation of many of the same inflammatory pathways identified here (e.g., TNF, IL-17, MAPK, NF-κB signaling, etc.) (65). They demonstrated that the actin cytoskeleton is involved in epithelial cell invasion, and inhibition of F-actin polymerization prevented Mcat invasion (65). These responses are also linked to NTHi-induced epithelial cell apoptosis (66). In our coinfection study, we saw that this effect was primarily induced via Mcat or Mcat secretome via ITGA5/SEPTIN1 (Fig. S4A), thereby promoting invasion for itself and potentially even accompanying NTHi.

Another study identified human biological processes from COPD patient blood transcriptomes (67), showing that heme metabolism, interferon-alpha, and interferon-gamma pathways (MSigdb) were affected in patients during both the presence and absence of COPD exacerbation, via different genes. We find some of the genes involved in interferon-alpha (19/97 genes in the pathway) and interferon-gamma pathways (31/200) in gene module H292_A. These include interferon-regulatory factors (IRFs) and their downstream genes, which are associated with the viral infection pathways enriched by this module. This suggests that Mcat—and NTHi to a lesser degree—can induce these exacerbation signatures at the epithelial cell level.

NTHi is capable of direct intracellular invasion in a strain-dependent manner (68). Using primary bronchial epithelial cells, NTHi infects various epithelial subtypes and induces inflammatory responses via TLR2/4 and downstream via IRF5/7, NOD1/2, and inflammasome-associated nucleotide-binding oligomerization domain-like receptors, some of which were also influenced by Mcat (69). We observed many of these responses at the pathway level in our study. We observed unchanged expression of CLND4 during NTHi infection, potentially allowing paracellular invasion, whereas Mcat induced its upregulation, suggesting that coinfection may cause preferential invasion of NTHi intracellularly (Fig. S4D). We also found upregulation of TLR2/4 and IRF7 relative to uninfected cells (Fig. S4D), and these were also enhanced during Mcat mono-infection and coinfection. NTHi invasion via NTHi LOS interaction with the PAFR receptor has been characterized (70); however, these genes were not significantly or consistently differentially expressed in our infection conditions with either species.

While we identified multiple biological processes related to NTHi and Mcat epithelium mono-/coinfection, we acknowledge certain limitations of our study. The use of H292 cells does not fully represent *in vivo* COPD conditions, nor does the impact of differentiated and ciliated epithelia or the influence of other species in the lung microbiome. However, ciliary function is impaired in COPD infections (71, 72). Our bacterial results in H292 cells are likely more relevant further into COPD infections, following bacteria-induced ciliary inhibition. Findings could be expanded in future studies using a transwell or organoid model of infection with primary, normalized human bronchial epithelial cells, mono/coinfected with NTHi and Mcat. Moreover, the transcriptional data described may not correlate with protein expression, and gene expression was not independently experimentally validated. Despite these, the study identifies bacterial and host avenues for future research and experimental validation, such as the preferred and detailed means of NTHi/Mcat transcellular epithelial invasion. Our studies also highlight areas for potential therapeutic intervention, such as dampening exacerbation severity by inhibiting Mcat-induced interferon responses, identifying bacterial protein vaccine candidates with unvarying elevated gene expression, or targeting inhibition of required bacterial nutrient uptake mechanisms. In conclusion, the data presented here provide valuable insights into NTHi and Mcat mono-/coinfections in COPD and their potential impacts on the disease and its research community.

## Data Availability

Transcriptomics data from this study were deposited at the Gene Expression Omnibus repository under accession no. GSE283527. Detailed information about methods and data availability, including genome accession numbers, bacterial strains, reagents, and software packages, is available in the Supplemental Material.

## SUPPLEMENTAL MATERIAL

Supplemental Material msphere.00242-25-s0001.pdf Supplemental text, Figures S1-S5, and captions for Tables S1 to S6.

Table S1 msphere.00242-25-s0002.xlsb Read mapping statistics for NTHi, Mcat, and human samples.

Table S2 msphere.00242-25-s0003.xlsx Differentially expressed gene lists for all NTHi comparisons (Fig. 3).

Table S3 msphere.00242-25-s0004.xlsx Differentially expressed gene lists for all Mcat comparisons (Fig. 3B).

Table S4 msphere.00242-25-s0005.xlsx Differentially expressed gene lists for all human comparisons (Fig. 3C).

Table S5 msphere.00242-25-s0006.xlsx Complete list of WGCNA gene module data and NTHi and Mcat biological pathways tested for significance.

Table S6 msphere.00242-25-s0007.xlsx Complete list of WGCNA gene module data for Human cells and enriched biological pathways (Fig. 4C).

ASM does not own the copyrights to Supplemental Material that may be linked to, or accessed through, an article. The authors have granted ASM a non-exclusive, world-wide license to publish the Supplemental Material files. Please contact the corresponding author directly for reuse.
