# Supplementary material for: Host-pathogen interaction profiling of nontypeable Haemophilus influenzae and Moraxella catarrhalis coinfection of bronchial epithelial cells
Source: mSphere. 2025 Jun 10;10(7):e00242-25. doi: 10.1128/msphere.00242-25 (PMC12306180; doi:10.1128/msphere.00242-25)
Supplement: Supplemental Material — Supplemental text, Figures S1-S5, and captions for Tables S1 to S6. [file msphere.00242-25-s0001.pdf]

Supplemental Materials

Methods

All transcriptomics data generated as a part of this study have been deposited at the Gene Expression Omnibus repository under accession no. GSE283527.

Bacterial strains

The NTHi and Mcat strains used in this study were isolated from the sputum of an adult with severe COPD (Forced Expired Volume in 1 second [FEV1] = 46% predicted) enrolled in a 20-year prospective study of adults with COPD that was conducted at the Buffalo Veterans Affairs Medical Center (1-3). NTHi strain 48P106H1 caused an exacerbation of COPD upon acquisition by the patient and then persisted in the airways for 993 days documented by monthly cultures of sputum. Mcat strain 48P118B was isolated from the sputum of the same person during the time that the NTHi strain was present in the airways. Mcat strain 48P118B was isolated from a single monthly sputum culture, indicating that it persisted for 30 days or less. The patient experienced an exacerbation simultaneous with acquisition of Mcat strain 48P118B. The patient experienced 5 additional exacerbations during the 993 days that NTHi strain 48P106H1 persisted.

In vitro cell culture models

Three independent sets of flasks were prepared for each condition as shown in Figure 1. Two days prior to the assay, NCI-H292 cells were grown to confluence in culture media, which contained RPMI 1640 medium (ATCC modification) and 10% heat inactivated fetal bovine serum (FBS). After 24 hours and when cells reached confluence, the culture media was replaced with base media (i.e., RPMI 1640 medium) and no FBS. Quantitative cultures of sputum during our prior 20 year prospective study indicated that NTHi and Mcat are present in cultures in varying concentrations from 10^5-10^9 cfu/ml, with majority of the samples with positive cultures showing 10^7-10^9 cfu/ml. Guided by these results, we targeted a ~1:1 proportion of NTHi:Mcat during inoculation. Low passage bacterial strains were grown on chocolate agar II plates and incubated overnight at 37°C with 5% CO_2. On the day of the assay, liquid bacteria cultures were started from a fresh overnight plate at OD600 ~0.1 and grown at 37°C, 250rpm to OD600 ~0.4 to 0.5 (approx. 4-5 x 10^8 CFUs). NTHi was cultured in brain heart infusion (BHI) broth + 5% Fildes and Mcat was grown in BHI broth. Bacteria were placed on ice, 6 ml of bacteria were centrifuged at 3,220 x g for 5 min, the pellet was washed with 6 mL D-PBS, pelleted again and suspended in in six mL base media (RPMI 1640). For the media alone assays (wo), we added 4mL of base media into new TC flasks with 2mL washed bacteria (2ml of NTHi or 2ml Mcat or 1mL each for the mix). The flasks were incubated for 5 hours at 37°C with 5% CO_2 based on technical feasibility and prior work in our labs (4). We previously performed experiments examining NTHi adherence and invasion of H292 cells at time points ranging from 1 to 6 hours. High levels of adherence after 1 hour and greater mean invasion at later time points were observed. However, there was a decline in intracellular survival over time. Hence, we estimated 5 hours would provide sufficient time for adherence, invasion, limit decline in intracellular survival, and allow for adaptation to environmental conditions and significant changes in the transcriptome. For the *in vitro* cell culture assays, the base media was removed from the flasks containing confluent H292 cells and replaced with 4 mL fresh base media per flask, two mL of washed bacteria were added per flask, the samples were mixed by gentle pipetting and the flasks incubated for 5 hours at 37°C with 5% CO_2. The NTHi and Mcat apical fluid flasks

(Msup, referred to as Mcat secretome) were prepared in a similar manner with minor modifications. Two mLs of Mcat were added to confluent H292 cells, after a 5-hour incubation at 37°C with 5% CO_2, the Mcat apical fluid was removed and filtered through a 0.22µm syringe filter and kept on ice. For the assay, 1.5 mL of Mcat apical fluid was added with 0.5 mL of 2X concentrated NTHi and the flasks were incubated for 5 hours at 37°C with 5% CO_2. Flasks containing infected H292 cells were fractioned into "apical fluid" (apf), containing free floating non-adhered/non-invaded bacteria, and adhered/invaded bacteria on or within H292 cells (cell). The fractions were transferred to RNAprotect (Qiagen, Valencia, CA). Samples were vortexed for 5 seconds, incubated for five minutes at room temperature and then centrifuged for 10 min at 5000 x g and 4°C. The supernatant was discarded, and the pellet frozen at -80°C until RNA extraction.

RNA extraction and sequencing

RNA was extracted using a pre-lysis step, the Maxwell 16 LEV simplyRNA Tissue Kit (Cat# AS1280), and the Maxwell 16 Instrument (Promega) according to the manufacturer's instructions. Briefly, the bacteria pellet was thawed and suspended in 200µL PBS with mutanolysin and proteinase K and incubated for 25 minutes at 37°C. We added 400µL homogenization solution containing 1-thioglycerol, vortexed the sample, put it on ice and added 400µL lysis buffer. Samples were vortexed and transferred to the maxwell LEV cartridge. After extraction, we added an extra DNAse treatment of the RNA with RNase-Free DNase (Qiagen #79254). RNA was purified using the Qiagen RNeasy Mini Elute Clean-up kit according to the manufacturer's instructions. 1µg of purified RNA was sent to the Yale Center for Genome Analysis (YCGA) for sequencing. The Kapa Biosystems rRNA depletion kit was used for ribosomal RNA reduction and samples were sequenced on the Illumina NovaSeq 6000 instrument using a 100bp paired-end read protocol and multiplexed with a target of 10 million reads per sample for bacteria apical fluid experiments, 5 million reads for bacteria culture, 75 million reads for H292 cells, and 150M reads per sample for bacteria/cell line culture experiments.

Transcriptomics and pathway analysis

FASTQ files were mapped to their respective genomes using HISAT for *Homo sapiens* and Bowtie2 for NTHi 48P106H1 and Mcat 48P118B genomes (Accession numbers NTHi: CP020006, Mcat: CP158365). Gene expression counts for all samples were estimated using HTseq followed by batch effect correction using CombatSeq, and estimation of differentially expressed (DE) genes using DESeq2. Principal Component Analyses (PCAs) and rarefaction curves were generated in R based on normalized Variance Stabilized Transformation (VST) counts acquired using the DESeq2 R package. For human DE gene estimation, singly infected and coinfected samples were compared to uninfected H292 control samples using DEseq2 and filtered using an FDR cutoff of ≤0.05 and an absolute Log2 Fold Change cutoff of ≥1. For bacterial DE gene estimation, each coinfected sample was compared to both singly infected samples in its respective media condition (in vitro media, host cell apical fluid, on the host cell) as well as comparisons across media condition (eg. host cell vs in vitro), using DEseq2 and filtered using an FDR cutoff of ≥0.05 and an absolute Log2 Fold Change cutoff of ≥1. Common and unique DE genes for both species were determined using Upset plots (R package UpsetR) and individual heatmaps of gene regulons and pathways were generated based on Z-scores of VST counts (R package DESeq2) using the R package 'pheatmap'. WGCNA was performed using the R package WGCNA, with all DE genes for each species having an average VST count >=7, using a softpower of 9, and default parameters.

Human DE gene lists were investigated using ShinyGO (5) to determine annotations and KEGG pathway enrichments, some of which were plotted using the R packages KEGGProfile (6) and Pathview (7). For bacterial pathways, gene expression pathways and regulons were acquired from 3 sources, namely, Kyoto Encyclopedia of Genes and Genomes (KEGG) (8), RegPrecise (9) and Virulence Factor Database (VFDB) (10). Some of these sources (RegPrecise & VFDB) were supplemented for Mcat genes using orthology of *Moraxella catarrhalis* 48P118B to *Acinetobacter baumannii* AB0057 RegPrecise, and literature of Mcat virulence factors (11). Significance for enriched pathways were determined for each gene expression module using hypergeometric tests in R.

Results

Bacterial and human transcriptional profiles reflect major differences between mono-infection and coinfection.

To assess the effect of coinfection on bacterial and human transcriptional profiles, we infected H292 cells with NTHi and Mcat individually and in coinfection (Mix) (Figure 1). To ensure sufficient bacterial RNA-Seq data, we performed transcriptomic quality control using rarefaction curves (Figure S1) for reads mapped to the genomes of both species (Accessions NTHi: CP020006, Mcat: CP158365). The rarefaction curves for NTHi and Mcat plateaued for all samples, indicating we had sufficient sequencing depth for quality downstream analysis of bacterial gene expression profiles. Another consideration when performing RNA-Seq with multiple species from the same domain is cross mapping of reads across species. To verify that no cross-species mapping had occurred, all samples were mapped to both reference genomes of NTHi and Mcat. For example, samples that did not contain NTHi but did contain Mcat, had only a few thousand reads detected when mapping to NTHi, and vice versa. This indicated that no sizeable cross-species mapping was occurring. A summary of read mapping statistics and raw read counts for all species in all samples is provided in Table S1. As H292 cells vastly outnumbered bacterial cells in the multispecies samples with >95% of reads originating from human transcripts (Table S1), human samples had excellent coverage and all rarefaction curves plateaued (not shown).

We performed Principal Component Analysis (PCA) for each species to verify quality clustering of sample replicates and identify changes in genome-wide transcriptional responses across conditions. For NTHi samples (Figure 2A), PC1 (~45% variance) indicated that the NTHi response is heavily influenced by H292 cells; NTHi and NTHi+Mcat (Mix) infected H292 cells are on the left and NTHi grown in cell culture medium (wo) or non-invaded/non-adhered bacteria in apf are on the right. PC2 (~19%) reveals some separation of coinfected samples from NTHi alone. Mix sample types (cell, apf, and wo) cluster away from their respective NTHi counterparts, suggesting a significant impact of Mcat on NTHi in all sample types. NTHi samples exposed to Mcat secretome (i.e., Msup) closely cluster with respective unexposed NTHi samples, suggesting only a minor impact of the Mcat secretome on the NTHi response.

Similarly, the PCA of Mcat samples (Figure 2B), PC1 (~63%) revealed that the Mcat response to adherence/invasion of H292 cells is different from Mcat alone or in the apical compartment. However, unlike the NTHi PCA (Figure 2A), no clear separation was seen between Mcat and Mcat+NTHi (Mix) infected H292 cells suggesting that Mcat is less affected by the presence of NTHi during H292 coinfection. PC2 (~11%) partially separates Mix_apf samples and clusters Mcat_wo samples independently. Given that Mcat_apf samples cluster more closely with Mix_wo/apf

samples, this suggests that Mcat responds to both species in a similar manner when in a planktonic state or doesn't interact much with host cells.

The PCA of H292 cellular responses (Figure 2C) PC1 (~44%) showed that all bacteria-infected samples were distinct from uninfected samples, as expected. Strikingly, there was separation observed among infection conditions, with Mcat infections closer to uninfected samples than NTHi containing samples, implying that Mcat infection induces a somewhat blunted response from H292 cells compared to NTHi. PC2 (~15%) separates Mcat-infected samples (bottom half) from NTHi-infected samples (top half). While all NTHi-containing samples are in the same vertical plane (along PC2), NTHi infections exposed to Mcat secretome form their own cluster implying a stronger response of H292 cells to the presence of NTHi and the Mcat secretome relative to NTHi alone.

NTHi, Mcat and Human H292 cells differentially regulate specific gene sets during adhered and invaded conditions.

We identified differentially expressed (DE) genes for all species across growth conditions/fractions, i.e. in media alone (wo), in (co)infected apical fluid (apf), and during epithelial cell adherence/invasion (cell) using DESeq2 (12). Several NTHi genes were DE (Figure 3A, top two horizontal bars) in all H292 adhered/invaded cells relative to media alone implying a robust response to direct contact with H292 cells. However, relative to apical fluid bacteria, fewer DE genes were observed (Figure 3A, next two bars), likely due to some exposure of NTHi to H292 cells indirectly in the apical fluid. Of these four comparisons, 86 NTHi genes were shared across all H292 adhered/invaded cells and not among the other sample types (apf & wo, Figure 3A, blue bar of shared genes). These genes primarily consisted of ribosomal genes, transcription/translation genes, membrane integrated genes, tRNA modification genes, regulators, and heme proteins (Table S2). When comparing all adhered/invaded cell cocultures to each other, fewer than 100 genes were DE for each condition, suggesting modest yet distinct differences in responses.

Adhered/invaded Mcat on H292 cells alone had ~70% more DE genes than NTHi alone (Figure 3B, top bar), indicating a robust Mcat response to contact with H292. This pattern was also seen when Mcat and NTHi were cocultured in media (Figure 3B, 5th orange bar), with Mcat having ~3X the number of DE genes when compared to NTHi (Figure 3A, 5th orange bar). Together these results indicate that Mcat produces a greater response to either organism it encounters in its environment than NTHi does. For Mcat, 83 genes were shared across all H292 adhered/invaded cells and not among the other conditions (Figure 3B, blue bar of shared genes). Of these, the vast majority encoded hypothetical proteins, while other genes included DNA modification genes, transcription/translation genes, diverse enzymes, and transporters (Table S3).

Lastly, for H292 DE genes, all infected conditions were compared to uninfected H292 cells. Thousands of H292 genes were DE across all infection conditions, with similar amounts for NTHi infection and NTHi/Mcat coinfection (Figure 3C, top 3 bars). Interestingly, Mcat infection regulated ~30% fewer H292 DE genes than the other conditions, (Figure 3C, bottom bar), suggesting a blunted response of H292 cells, a pattern that was also observed on the H292 PCA (Figure 2C). Overall, 3,240 H292 genes were DE across all infection conditions (Figure 3C, blue bar of shared genes). Of these, approximately half were protein coding genes, a quarter were long non-coding RNAs (lncRNAs), and the rest were other non-protein coding genes. Some of the protein coding genes included host H292 infection genes such as interleukins, chemokines, interferons, MHCs, NF-κB, and TNF related genes. Descriptions of all these genes are provided in Table S4.

A further breakdown of all DE genes and their overlaps with other conditions is provided in the form of Upset plots in Figure S2, for each species. DE gene lists for each comparison are also listed in Tables S2 (NTHi), S3 (Mcat) and S4 (H292).

We performed WGCNA-based clustering of the normalized expression values of all DE genes (after filtering, see Methods) for each species to identify specific gene expression patterns that correlated with certain sample combinations (e.g., NTHi_cell, Mcat_cell, etc). Multiple gene expression modules, defined as subsets of DE genes with similar expression profiles, were detected for each species.

For NTHi (Figure 4A), the largest module consisted of 337 genes and was directly associated with NTHi invaded/adhered to H292 cells, regardless of the presence of Mcat secretome or Mcat coinfection (module NTHi_A). Genes in this module conformed to the same expression pattern in all samples with H292 cells (NTHi_cell, NTHi_Msup_cell and Mix_cell) are enclosed in black boxes to exemplify similar expression trends. These included 66 of the 86 genes that were DE in invaded/adhered NTHi relative to other samples (blue bars, Figure 3A). There were 7 other modules (NTHi B through H) that also correlated with certain sample types for NTHi. Module NTHi_B harbored genes with enhanced regulation for NTHi individual culture in media (NTHi_wo). Module NTHi_C primarily contained upregulated genes specific to NTHi apical fluid during co-culture on H292 cells (Mix_apf). This suggests that when NTHi is in apical fluid during coinfection, it follows a specific gene regulatory pattern that prevents invasion/adherence or enables survival at the apical surface. Module NTHi_D consisted of genes specific to coinfection of H292 cells at any stage of H292 infection (Mix_cell and Mix_apf). Module NTHi_E selected for NTHi genes responsive to Mcat secretome only during invasion/adhesion (NTHi_Msup_cell) and not during apical survival (NTHi_Msup_apf). Module NTHi_F highlighted genes that are downregulated during coinfection of H292 cells at any stage of H292 infection (Mix_cell and Mix_apf), suggesting that Mcat presence supplements their usage. Modules NTHi_G and NTHi_H consisted of NTHi genes that were particularly sensitive to the Mcat secretome (NTHi_Msup_cell/NTHi_Msup_apf).

Fewer modules of regulated genes were detected for Mcat compared to NTHi (Figure 4B). Two modules, Mcat_A and Mcat_B, reflected gene expression changes driven by Mcat in individual culture in media (Mcat_wo, similar to module NTHi_B). This is reminiscent of the clustering of the Mcat_wo samples far from other conditions on the PCA (Figure 2B). Similar to module NTHi_A, Mcat modules Mcat_A (444 genes) and Mcat_B (252) were directly associated with Mcat invaded/adhered to H292 cells and not apf (Mcat_cell and Mix_cell), regardless of the presence of NTHi in coinfection. They included 65 (48 of Mcat_A and 17 of Mcat_B) of the 83 genes that were DE in invaded/adhered Mcat relative to other samples (blue bars, Figure 3B). Module Mcat_C genes also partially associated with Mcat invaded/adhered to H292 cells and not apf, albeit with variation among some replicates (Mcat_cell and Mix_cell). Module Mcat_D and Mcat_F showcased small gene sets that were upregulated by Mcat during invasion/adherence during coinfection (Mix_cell). Lists of all the genes in NTHi and Mcat modules are in Table S5.

Five major modules were detected for H292 cells. Three of the 5 modules (not shown) consisted of genes that were differentially regulated across all infection conditions relative to uninfected H292 cells; these modules did not clearly distinguish gene expression patterns specific to NTHi, Mcat, or mixed infection. They consisted of 4,421 genes of which 1,481 were DE in infected H292 cells vs uninfected (Figure 3C, blue bars). However, two modules H292_A and H292_B did highlight gene expression patterns specific to Mcat

presence (Mcat_cell and Mix_cell), as well as specific to NTHi presence alone (NTHi_cell and NTHi_Msup_cell), respectively (Table S6).

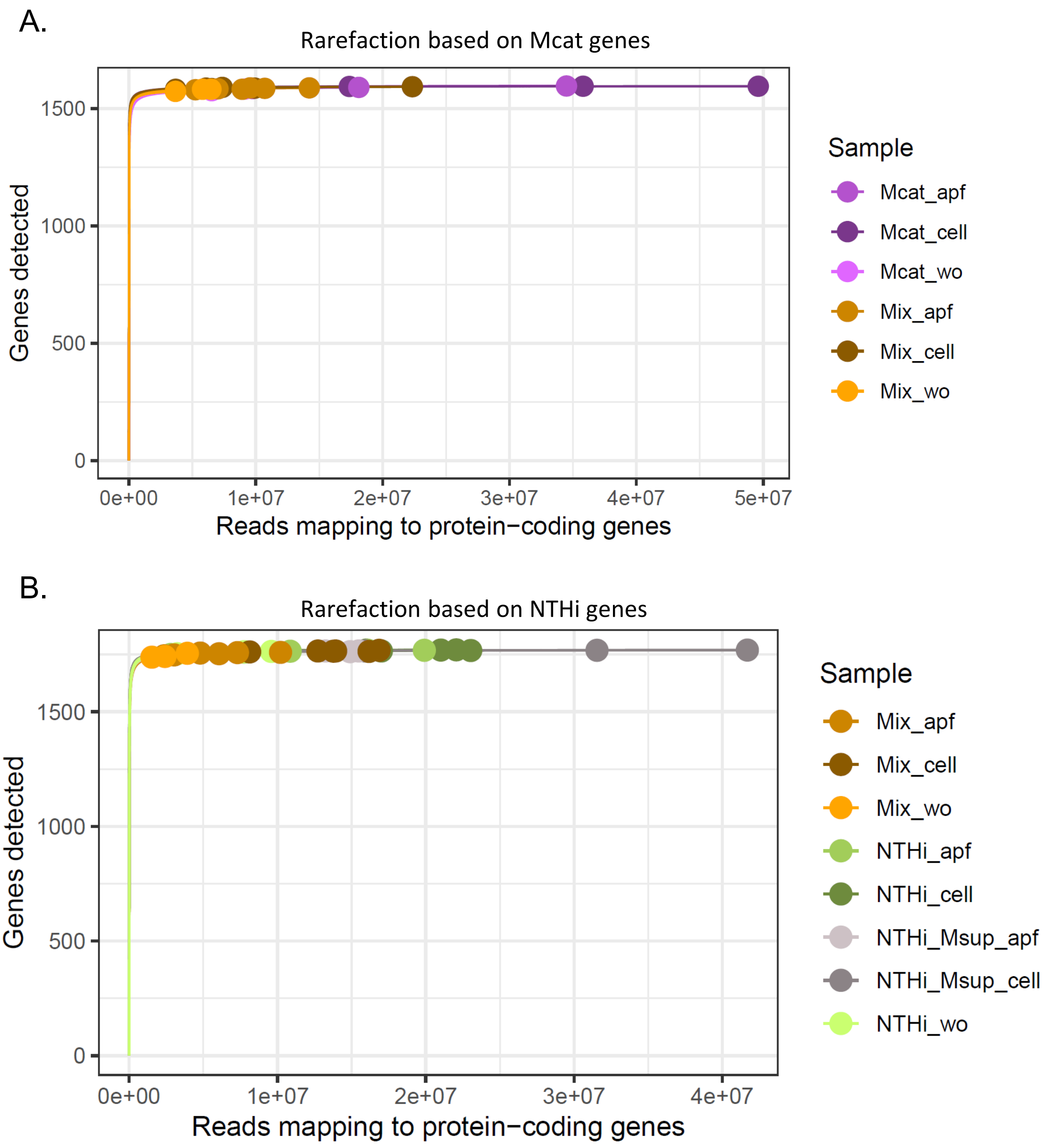

Figure S1. Rarefaction curves of RNA-seq reads mapped to their respective NTHi or Mcat genome sequences. RNA-seq samples with gene detection curves that plateaued indicate sufficient read coverage across their genome. A) Rarefaction curve based on 1,610 Mcat genes. B) Rarefaction curve based on 1,774 NTHi genes. Mapping statistics for all individual samples are provided in Supplemental Table 1.

Figure S2. Upset plots of differentially expressed (DE) genes. Expanded plots of all DE genes showing various shared intersections of DE genes between different comparisons for A) NTHi B) Mcat and C) Human

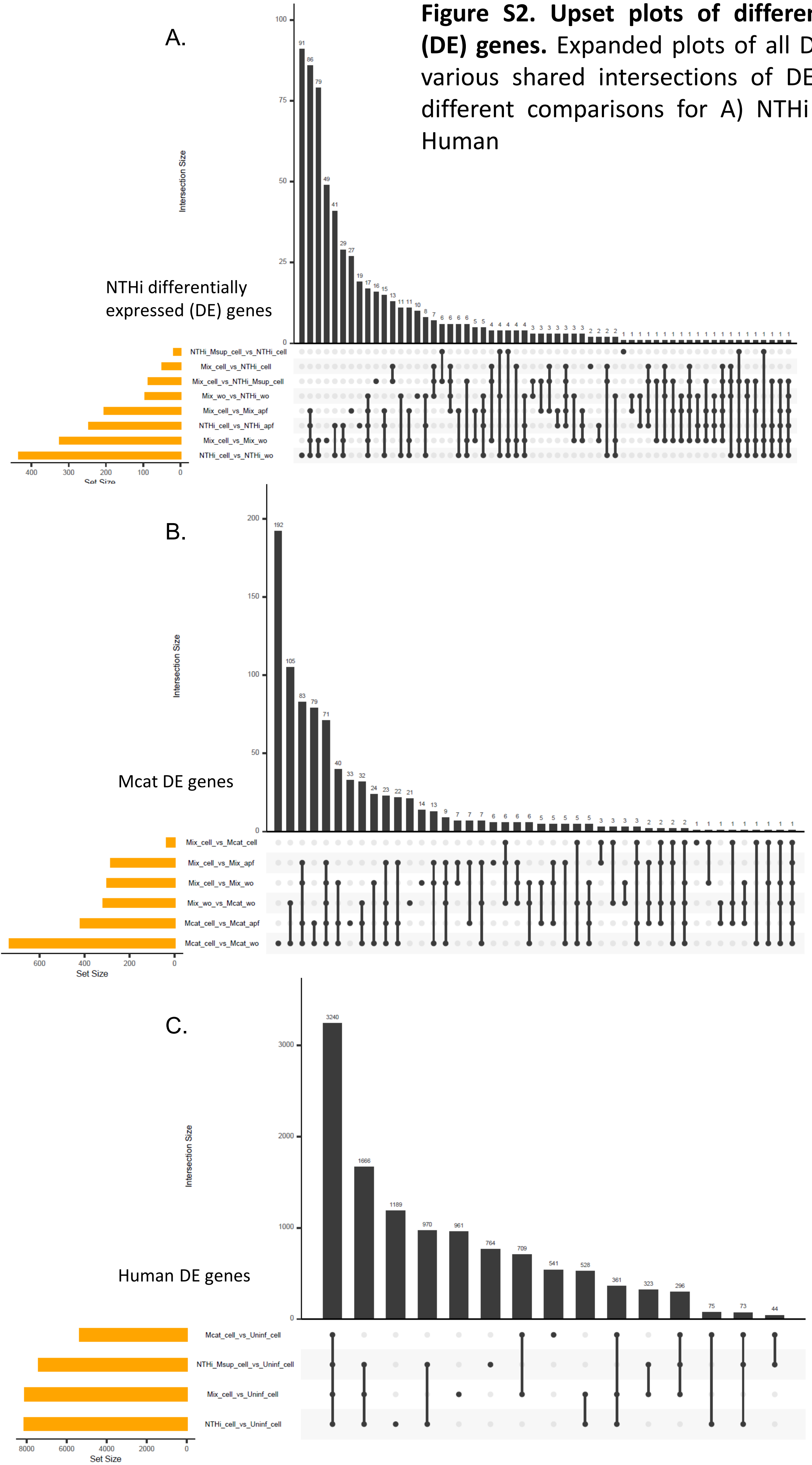

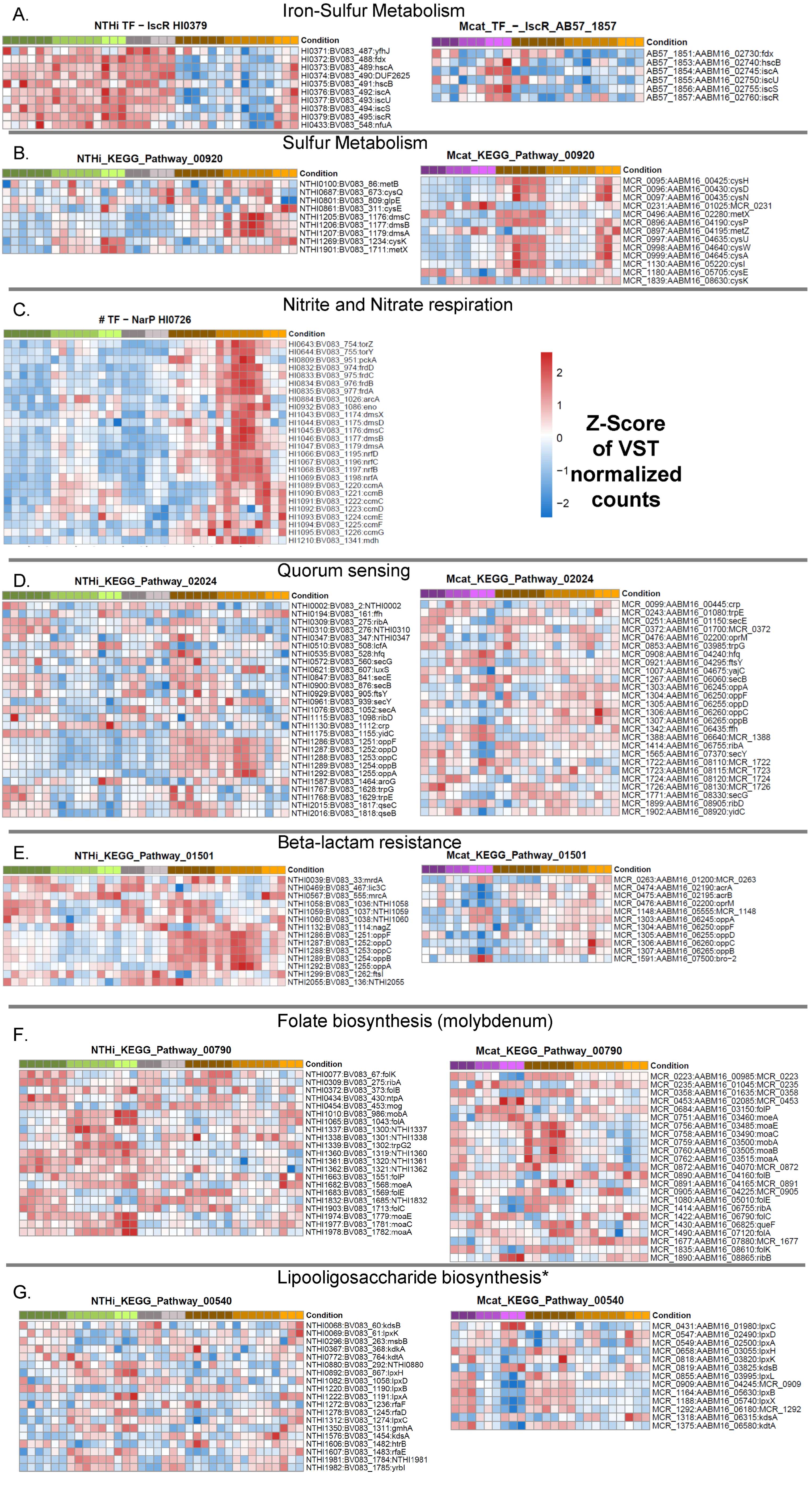

Figure S3. Z scored heatmaps of genes in significantly enriched bacterial pathways from Figure 5. Heatmaps are shown for enriched pathways of either NTHi (left) or Mcat (right) wherever available to highlight differences in regulation between the two species. *Not significantly enriched.

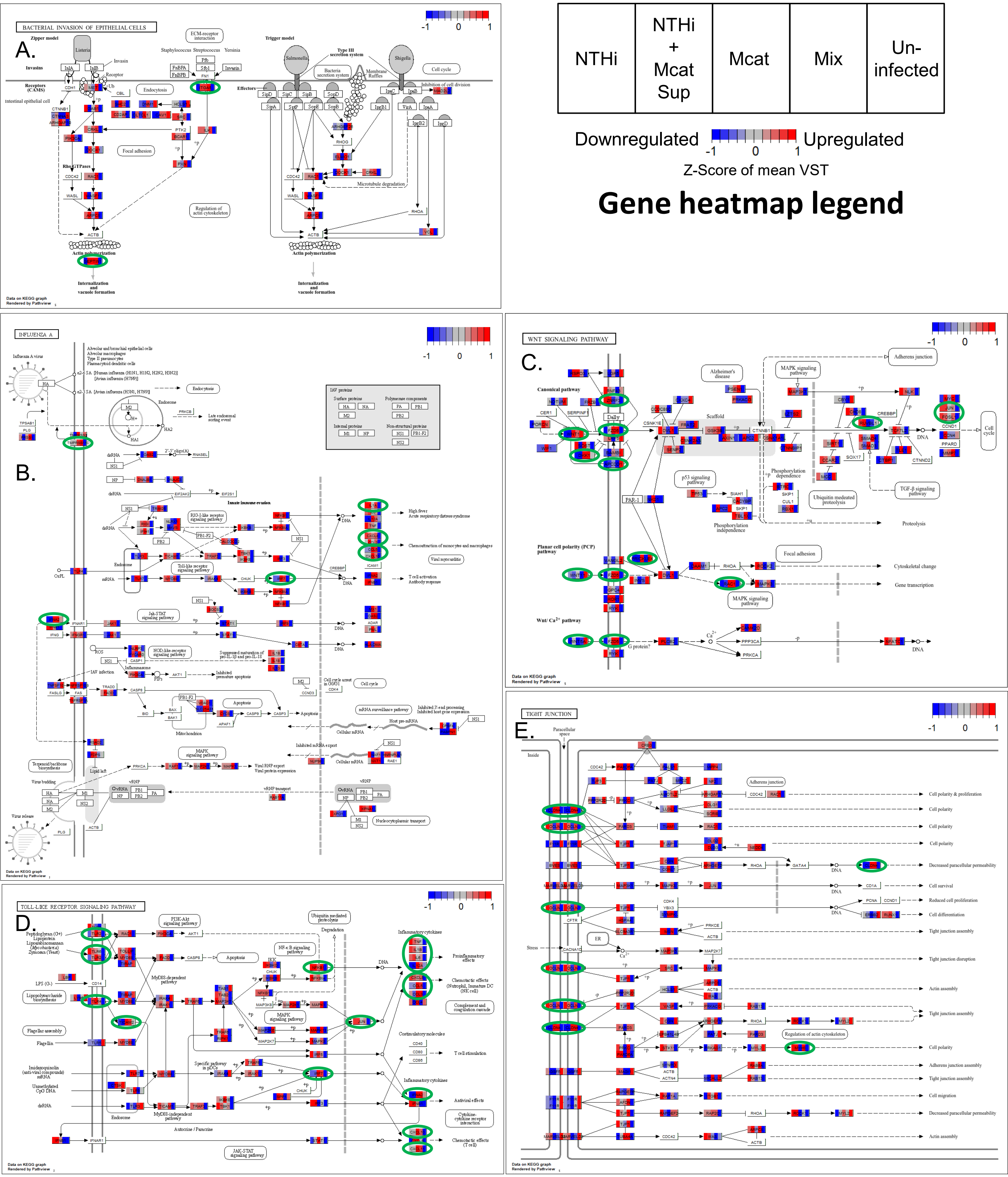

Figure S4. Z scored heatmaps of individual genes in significantly enriched human pathways. Heatmaps are shown for each gene of 5 enriched human pathways wherever available. Specific genes that have consistent regulation across NTHi infected (NTHi, NTHi+Mcat sup, Mix) or Mcat infected (Mcat, Mix) samples, are discussed in text and are highlighted in green ellipses.

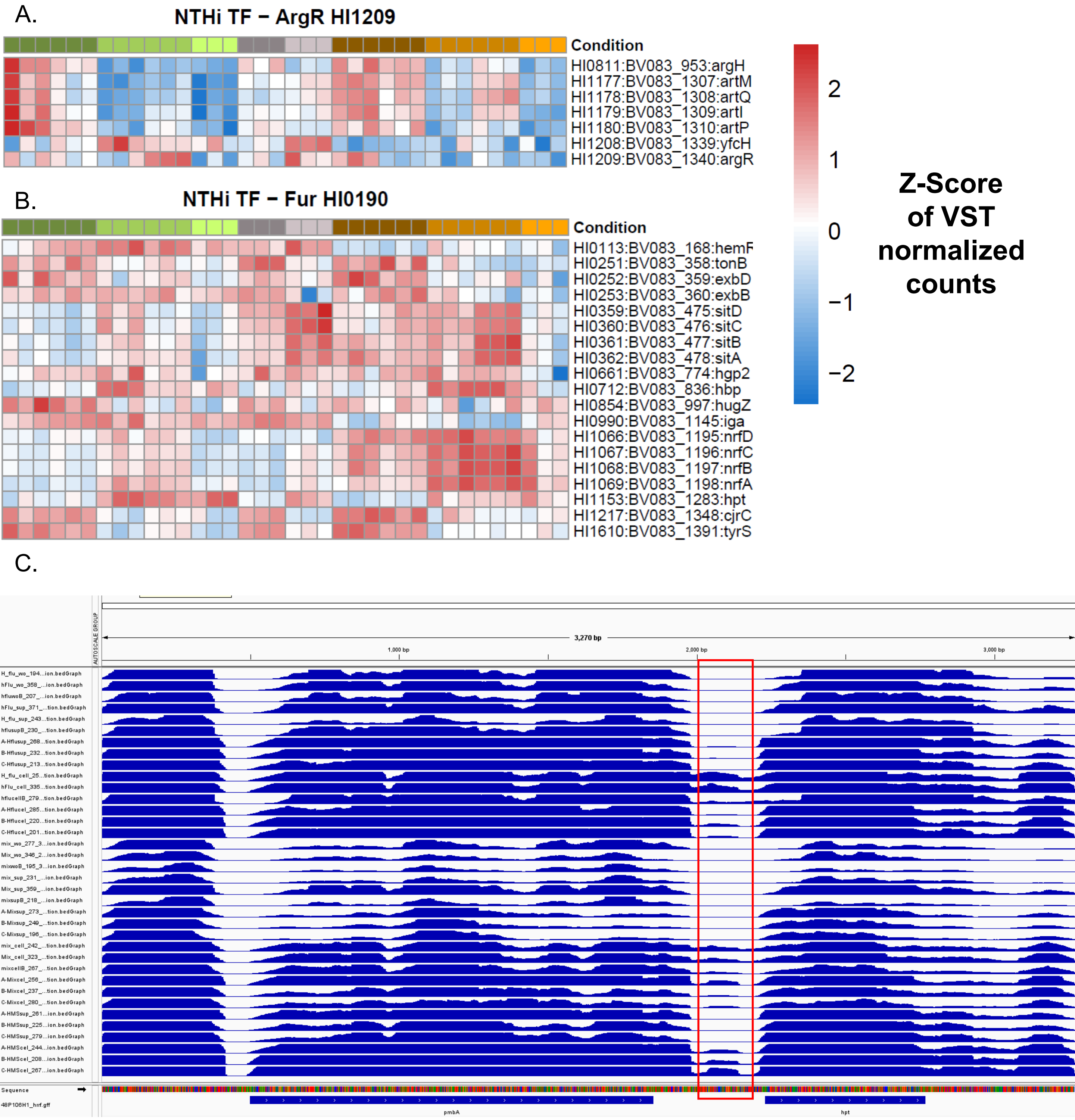

Figure S5. NTHi ArgR and Fur regulons . A) Z scored heatmap of NTHi ArgR regulon. B) Z scored heatmap of NTHi Fur regulon. C) RNA-seq coverage (visualized in IGV) of the NTHi HrrF sRNA regulator region (red box), primarily seen on host cells, and the apical surface to a much lesser degree.

Supplemental Dataset Legends

Table S1: Read mapping statistics for NTHi, Mcat & Human samples. Raw (HTseq) read count tables for NTHi, Mcat & Human samples.

Table S2: Normalized (VST) read counts and differentially expressed gene lists for all NTHi comparisons (Figure 3A).

Table S3: Normalized (VST) read counts and differentially expressed gene lists for all Mcat comparisons (Figure 3B).

Table S4: Normalized (VST) read counts and differentially expressed gene lists for all Human comparisons (Figure 3C).

Table S5: Complete list of NTHi and Mcat biological pathways tested for significance. WGCNA gene module data for NTHi and Mcat (Figure 4A, 4B). Significantly enriched biological pathways for each module of NTHi and Mcat (Figure 6A, 6B)

Table S6: WGCNA gene module data for Human cells (Figure 4C). Significantly enriched biological pathways for each module of Human cells (Figure 6C and more pathways).

Supplemental Materials References

1. Sethi S, Evans N, Grant BJB, Murphy TF. New strains of bacteria and exacerbations of chronic obstructive pulmonary disease. N Engl J Med. 2002;347:465-71.
2. Murphy TF, Brauer AL, Grant BJ, Sethi S. *Moraxella catarrhalis* in chronic obstructive pulmonary disease. Burden of disease and immune response. Am J Respir Crit Care Med. 2005;172:195-9.
3. Pettigrew MM, Ahearn CP, Gent JF, Kong Y, Gallo MC, Munro JB, et al. Haemophilus influenzae genome evolution during persistence in the human airways in chronic obstructive pulmonary disease. Proc Natl Acad Sci U S A. 2018;115(14):E3256-E65. doi: 10.1073/pnas.1719654115. PubMed PMID: 29555745; PubMed Central PMCID: PMC5889651.
4. Ahearn CP, Kirkham C, Chaves LD, Kong Y, Pettigrew MM, Murphy TF. Discovery and Contribution of Nontypeable Haemophilus influenzae NTHI1441 to Human Respiratory Epithelial Cell Invasion. Infect Immun. 2019;87(11). Epub 20191018. doi: 10.1128/IAI.00462-19. PubMed PMID: 31427451; PubMed Central PMCID: PMC6803334.
5. Ge SX, Jung D, Yao R. ShinyGO: a graphical gene-set enrichment tool for animals and plants. Bioinformatics. 2020;36(8):2628-9. doi: 10.1093/bioinformatics/btz931. PubMed PMID: 31882993; PubMed Central PMCID: PMC7178415.
6. Shilin Zhao YG, and Y Shyr. Keggprofile: An annotation and visualization package for multi-types and multi-groups expression data in kegg pathway. R package version, 1(1), 2012. 2012.
7. Luo W, Brouwer C. Pathview: an R/Bioconductor package for pathway-based data integration and visualization. Bioinformatics. 2013;29(14):1830-1. Epub 20130604. doi: 10.1093/bioinformatics/btt285. PubMed PMID: 23740750; PubMed Central PMCID: PMC3702256.
8. Kanehisa M, Furumichi M, Sato Y, Kawashima M, Ishiguro-Watanabe M. KEGG for taxonomy-based analysis of pathways and genomes. Nucleic Acids Res. 2023;51(D1):D587-d92. doi: 10.1093/nar/gkac963. PubMed PMID: 36300620; PubMed Central PMCID: PMC9825424.
9. Novichkov PS, Kazakov AE, Ravcheev DA, Leyn SA, Kovaleva GY, Sutormin RA, et al. RegPrecise 3.0--a resource for genome-scale exploration of transcriptional regulation in bacteria. BMC Genomics. 2013;14:745. Epub 20131101. doi: 10.1186/1471-2164-14-745. PubMed PMID: 24175918; PubMed Central PMCID: PMC3840689.
10. Liu B, Zheng D, Zhou S, Chen L, Yang J. VFDB 2022: a general classification scheme for bacterial virulence factors. Nucleic Acids Res. 2022;50(D1):D912-D7. doi: 10.1093/nar/gkab1107. PubMed PMID: 34850947; PubMed Central PMCID: PMC8728188.
11. Blakeway LV, Tan A, Peak IRA, Seib KL. Virulence determinants of Moraxella catarrhalis: distribution and considerations for vaccine development. Microbiology (Reading). 2017;163(10):1371-84. Epub 20170912. doi: 10.1099/mic.0.000523. PubMed PMID: 28893369.
12. Love MI, Huber W, Anders S. Moderated estimation of fold change and dispersion for RNA-seq data with DESeq2. Genome Biol. 2014;15(12):550. doi: 10.1186/s13059-014-0550-8. PubMed PMID: 25516281; PubMed Central PMCID: PMC4302049.
